# Targeting a Shared Mitophagy Regulator: The SIRT1–FOXO3–DEPP1 Axis Underpins the Dual Bone and Brain Benefits of Total Flavonoids from *Drynaria fortunei*

**DOI:** 10.34133/research.1125

**Published:** 2026-02-24

**Authors:** Yili Zhang, Xiangyun Guo, Qing Wang, Long Xiao, Qingqing Liu, Yiwen Gan, Yunning Li, Chuanrui Sun, Zhiwen Luo, Kai Sun, Weiwei Tao, Xu Wei

**Affiliations:** ^1^ School of Integrated Chinese and Western Medicine, Nanjing University of Chinese Medicine, Nanjing 210023, Jiangsu, China.; ^2^ Department of Orthopaedics, Kunshan Hospital of Chinese Medicine, Suzhou 215300, Jiangsu, China.; ^3^ Translational Medical Innovation Center, Zhangjiagang TCM Hospital Affiliated to Nanjing University of Chinese Medicine, Suzhou 215600, Jiangsu, China.; ^4^ School of Medicine, Nanjing University of Chinese Medicine, Nanjing 210023, Jiangsu, China.; ^5^ Wangjing Hospital, China Academy of Chinese Medical Sciences, Beijing 100102, China.; ^6^ Department of Sports Medicine, Huashan Hospital, Fudan University, Shanghai 200040, China.

## Abstract

Postmenopausal osteoporosis and depression often occur together, but a single treatment that improves both conditions is currently lacking. The loss of estrogen can trigger oxidative stress, damage mitochondria, and drive dysregulated autophagy with impaired flux, simultaneously harming bone and the brain. We evaluated whether total flavonoids from *Drynaria fortunei* (TFDF) could counter these problems by activating sirtuin-1 (SIRT1), a protein that supports autophagy and mitochondrial health. In menopausal and chronic stress model mice and in cultured bone-forming cells and hippocampal neurons exposed to oxidative injury, we measured bone structure and strength indicators, mood-related behaviors, mitochondrial function, and gene activity patterns. The flavonoids preserved bone density and fine bone structure, shifted bone turnover toward formation, and improved depression-like behaviors (greater exploration and sucrose preference, less immobility). Across bone and the brain, TFDF modulated SIRT1–FOXO3–DEPP1 signaling and FOXO-linked oxidative stress and autophagy programs, thereby normalizing autophagic recycling and mitochondrial function. In cellular models, TFDF preserved mitochondrial function and restored autophagic recycling, and the loss or gain of SIRT1 function abolished or enhanced these benefits, respectively, indicating that SIRT1 activity is necessary for the effects of TFDF. These findings identify TFDF as a single, mechanism-based strategy that addresses both skeletal deterioration and depressive symptoms after menopause by engaging SIRT1-dependent stress autophagy pathways to restore cellular recycling and energy control.

## Introduction

Osteoporosis (OP) and depression frequently co-occur in postmenopausal women, suggesting a shared biological basis rather than a chance overlap [1,2]. Menopausal estrogen withdrawal is a common driver of both conditions: loss of estrogen accelerates bone resorption and skeletal microarchitectural deterioration while concurrently depriving the brain of the neurotrophic support needed for mood stability [3,4]. Increasing evidence points to convergent cellular processes underlying this bone–brain connection, including mitochondrial dysfunction, oxidative stress, and dysregulated autophagy/mitophagy with impaired flux, which together compromise the viability of bone and neural cells [5,6]. This results in a mutually reinforcing cycle in which skeletal pain and frailty worsen stress and depressive symptoms, while chronic stress hormones and proinflammatory mediators in turn accelerate bone loss [7]. This intersection of bone and brain pathology highlights a clear need for therapies that can simultaneously address both aspects of postmenopausal health [8].

Current treatment options remain compartmentalized and often at odds with one another. Antiresorptive and anabolic drugs for OP can reduce fracture risk but do little for mood or cognitive symptoms, as they are designed and evaluated primarily for skeletal endpoints [9–11]. Hormone replacement therapy can benefit both bone and mood, but its long-term use is limited by increased risks of cardiovascular events and hormone-sensitive cancers [12,13]. Even selective estrogen receptor modulators (SERMs) that improve bone density have minimal antidepressant effects and carry their own adverse profiles [14]. In short, there is currently no single approved agent that safely and effectively treats both skeletal degeneration and depressive disorder in postmenopausal women. An integrated therapeutic strategy is needed to break this cycle of bone loss and depression.

The total flavonoids from *Drynaria fortunei* (TFDF) is a standardized extract rich in osteogenic and neuroactive compounds, notably the flavonoids naringin and neoeriocitrin [15,16]. Preclinical studies indicate that these constituents exert parallel benefits on bone and the brain. In estrogen-deficient rodents, naringin prevents bone loss by stimulating osteoblast activity and suppressing osteoclasts, whereas in chronic stress models, it promotes adult hippocampal neurogenesis and alleviates depression-like behaviors [17–19]. Such dual osteoprotective and neuroprotective actions position TFDF as a plausible single agent capable of simultaneously addressing coexisting OP and depression [20]. However, to date, TFDF has not been evaluated in a model of combined OP and depression, and the common pathological mechanism linking these 2 disorders remains unclear. This knowledge gap necessitates investigating whether TFDF can simultaneously combat bone loss and mood dysfunction and by what molecular pathways.

To address this gap, we established a mice model combining ovariectomy and chronic unpredictable mild stress (OVX–CUMS) to mimic postmenopausal OP with concurrent depression-like behavior [21]. This integrated model allowed us to assess bone deterioration and depressive behaviors in the same animals. Using transcriptome-wide analysis of hippocampal and bone tissues, we sought to identify shared molecular disturbances in OVX–CUMS animals, with a particular focus on oxidative stress and autophagy pathways, and identified the stress-inducible autophagy regulator DEPP1 as a candidate shared node of disturbed autophagic homeostasis in bone and the hippocampus. On this basis, we hypothesized that TFDF would normalize DEPP1 expression and other stress–autophagy signatures in parallel with preventing bone loss and improving depressive-like behavior in this comorbid model. Given prior evidence that sirtuin-1 (SIRT1) and FOXO3 together orchestrate autophagy and mitochondrial quality control, we examined SIRT1 as a candidate upstream regulator of the shared bone and brain responses to TFDF [22]. SIRT1 is an NAD^+^-dependent deacetylase that enhances cellular stress resistance and is known to support bone formation and neuroplasticity [23,24]. Importantly, both estrogen deficiency and chronic stress are associated with reduced SIRT1 activity in bone and hippocampal tissues, which may exacerbate the vulnerability of osteoblasts and neurons [25,26]. We hypothesized that TFDF exerts its dual protective effects by engaging a SIRT1–FOXO3–DEPP1 signaling axis, thereby normalizing autophagic flux and mitochondrial function in both bone and the brain.

## Results

### TWAS prioritizes the hippocampus in OP–MDD comorbidity

Integrative transcriptome-wide association study (TWAS) (cross-tissue and S-MultiXcan) converged on the hippocampus as a shared susceptibility region for OP and major depressive disorder (MDD) after multiple-testing correction (false discovery rate < 0.05), with weaker or nonsignificant signals in most nonhippocampal regions. Tissue enrichment and partitioned heritability analyses further supported the presence of hippocampal involvement (Table S2). Guided by these human genetic findings, we next used the OVX–CUMS comorbidity model to validate alterations in both hippocampal and bone tissues and to assess whether TFDF could modulate shared cross-system mechanisms.

### TFDF ameliorates depressive-like behaviors and skeletal deterioration in OVX–CUMS mice

**Fig. 1.**
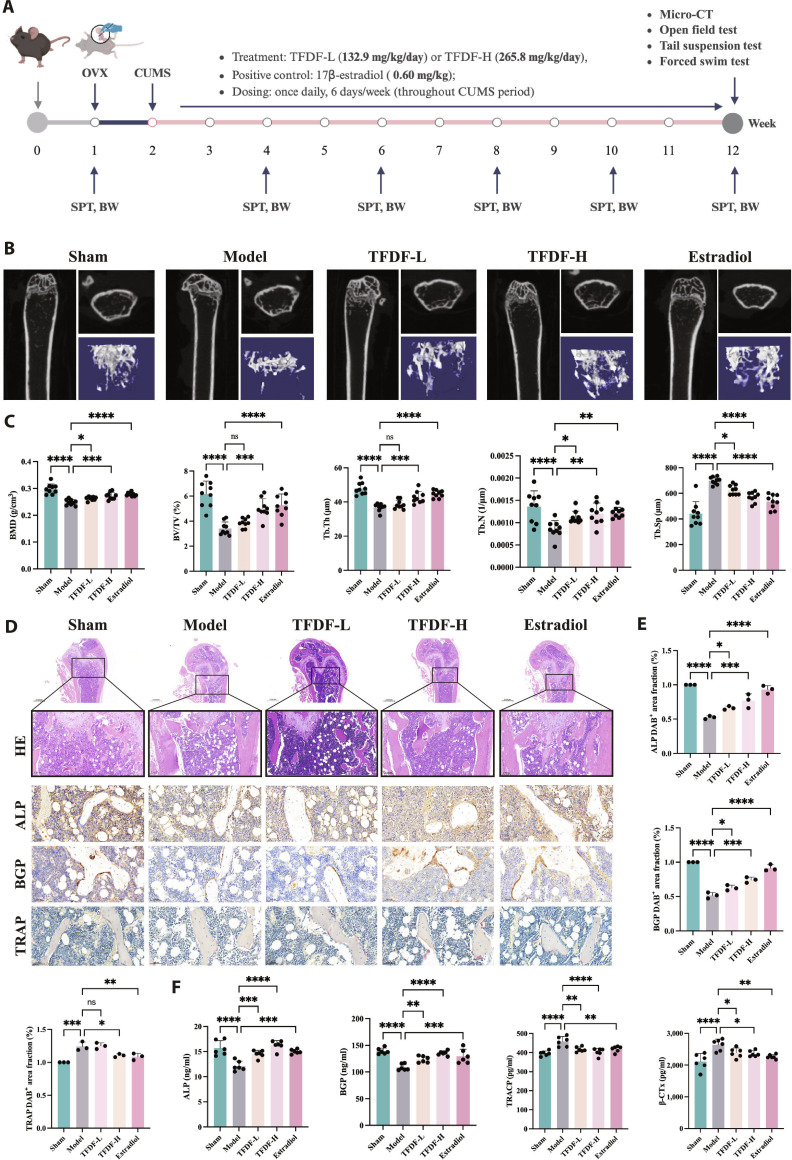
TFDF alleviates bone deterioration in OVX–CUMS mice. (A) Experimental design and grouping (Sham, OVX–CUMS model, TFDF-L, TFDF-H, and E2). TFDF, total flavonoids of *Drynaria fortunei*; low and high doses are indicated in Materials and Methods; E2, 17β-estradiol positive control. (B) Representative μCT images (2D slices and 3D reconstructions) of trabecular bone showing severe rarefaction in OVX–CUMS mice and preservation under TFDF/E2. (C) Quantitative μCT parameters: bone mineral density (BMD), bone volume/total volume (BV/TV), trabecular number (Tb.N), trabecular thickness (Tb.Th), and trabecular separation (Tb.Sp). (D) Histology and enzyme/immunostaining of the distal femur: hematoxylin and eosin for trabecular morphology; ALP (osteoblast activity), BGP/OCN (osteocalcin; osteoblast-derived), and TRAP (osteoclasts). (E) Quantification of staining: ALP-positive area, BGP/OCN-positive area, and TRAP-positive area. (F) Serum bone-turnover ELISAs for 2 formation markers and 2 resorption markers. ns, *P* ≥ 0.05; *P < 0.05; **P < 0.01; ***P < 0.001; ****P < 0.0001 vs. indicated group.

Guided by the TWAS results, we first established an in vivo comorbid phenotype and tested the protective effects of TFDF in the OVX–CUMS model; the study timeline and group allocation are shown in the schematic. Micro-computed tomography (micro-CT) imaging revealed pronounced trabecular rarefaction in OVX–CUMS mice compared with Sham mice, whereas TFDF preserved both the 2-dimensional architecture and 3-dimensional connectivity of trabecular networks at both tested doses, with the high dose approaching the 17β-estradiol (E2) reference (Fig. 1B). Quantitatively, OVX–CUMS reduced bone mineral density (BMD), bone volume fraction (BV/TV), trabecular number (Tb.N), and trabecular thickness (Tb.Th) but increased trabecular separation (Tb.Sp); compared with the Model group, the TFDF group increased BMD, BV/TV, Tb.N, and Tb.Th and decreased Tb.Sp, with the high-dose group showing the greatest recovery (Fig. 1C). Histology corroborated these findings: hematoxylin and eosin (H&E) sections from OVX–CUMS mice displayed thinned and perforated trabeculae together with expanded marrow spaces, whereas TFDF-treated bones exhibited denser and better connected trabeculae (Fig. 1D, HE). Osteoblast-associated staining increased after TFDF (alkaline phosphatase [ALP] and bone Gla protein [BGP]/osteocalcin [OCN]), whereas tartrate-resistant acid phosphatase (TRAP) staining decreased, as shown qualitatively (Fig. 1D, ALP/BGP/TRAP) and by quantitative image analysis (ALP-positive and BGP-positive areas increased; the TRAP-positive area decreased) (Fig. 1E). Consistently, serum bone turnover assays revealed a shift toward formation: the expression levels of formation markers (for example, ALP and BGP/OCN) were elevated, and the expression levels of resorption markers (for example, TRAP 5b and ICTP/CTX I) were reduced in the TFDF groups compared with those in the OVX–CUMS group, with the high-dose group closest to the Sham/E2 group (Fig. 1F).

**Fig. 2.**
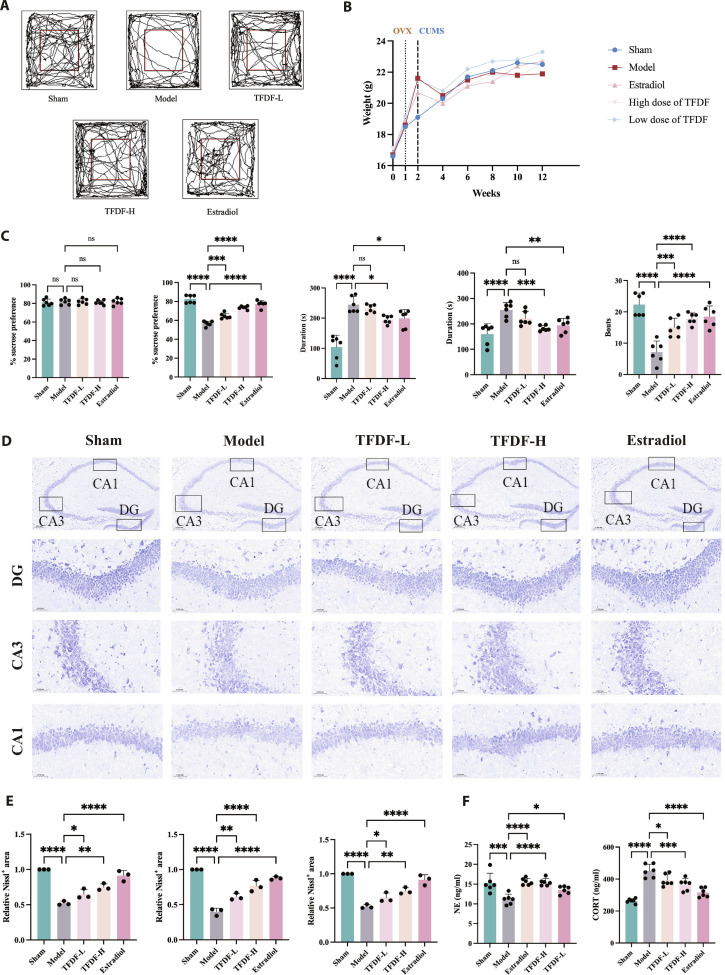
TFDF improves depression-like behaviors and preserves hippocampal neurons in OVX–CUMS mice. (A) Open-field (OFT) representative track plots for each group (Sham, OVX–CUMS model, TFDF-L, TFDF-H, and E2). (B) Body-weight trajectories during the intervention period. (C) Behavioral statistics: sucrose preference test (SPT, % sucrose preference), tail suspension test (TST, immobility time), forced swim test (FST, immobility time), and open field test (OFT, number of crossings in the central zone). (D) Hippocampal Nissl staining (DG/CA regions); representative images. (E) Quantification of Nissl-positive neurons/areas in the indicated subfields. (F) Serum ELISA results for corticosterone (CORT) and norepinephrine (NE).

Representative open-field trajectories illustrate the behavioral phenotype across groups (Fig. 2A): OVX–CUMS mice displayed curtailed exploratory paths, whereas TFDF increased exploration in a dose-responsive fashion and approached the E2 reference. Body-weight trajectories were recorded to contextualize behavior (Fig. 2B). Quantitatively, OVX–CUMS animals displayed a robust depression-like profile, with reduced sucrose preference, increased immobility in the tail suspension and forced swim tests, and diminished open-field locomotor and exploratory activity. TFDF attenuated these behavioral abnormalities across multiple measures (Fig. 2C). Hippocampal Nissl staining revealed decreased neuronal density and reduced Nissl substance in the dentate gyrus and CA subfields of OVX–CUMS mice (Fig. 2D). In TFDF-treated groups, neuronal morphology and layering were better preserved. Quantitative analysis confirmed higher Nissl-positive area and/or cell counts in the TFDF groups than in the model group (Fig. 2E). Endocrine readouts showed that serum corticosterone concentrations were reduced by TFDF compared with the model group, whereas norepinephrine levels showed a trend toward normalization (Fig. 2F).

### TFDF reverses a shared stress–autophagy signature in bone and the hippocampus and highlights DEPP1 as a common node

**Fig. 3.**
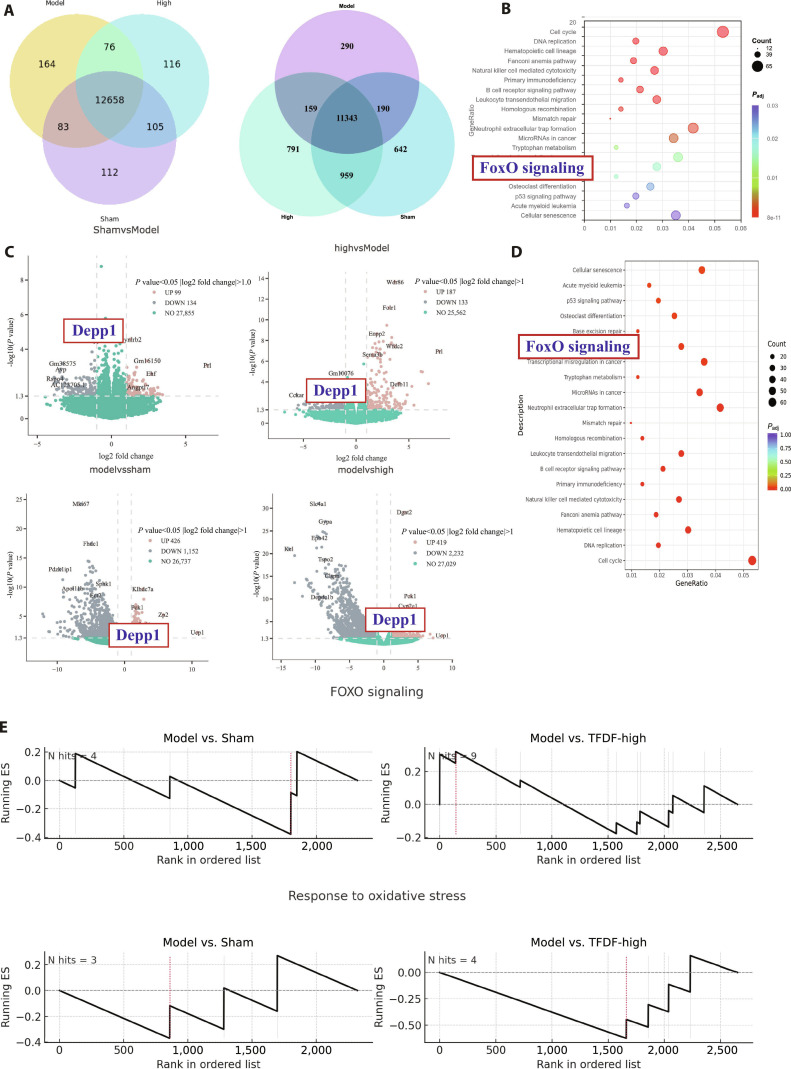
TFDF reverses a cross-tissue stress–autophagy signature and highlights DEPP1 as a shared node. (A) Venn diagram of TFDF-reversal DEGs in bone and the hippocampus (reversal defined as Model vs. Sham significant change, directionally opposed in TFDF-H vs. Model). (B) Pathway enrichment of reversal genes highlighting FOXO signaling (database and statistics in Materials and Methods). (C) Cross-filtering for Model↑ and TFDF-H↓ genes in both tissues identifies DEPP1 as a shared, treatment-reversed transcript (expression changes shown for bone and hippocampus). (D) Complementary enrichment of the reversal sets prioritizes FOXO signaling in both tissues. (E) GSEA plots demonstrating negative enrichment of FOXO programs in the Model vs. Sham groups and a positive shift in TFDF-H vs. Model (bone and hippocampus) groups.

Bulk RNA sequencing (RNA-seq) was performed on bone and the hippocampus from the Sham, OVX–CUMS (Model), and TFDF-H groups. Differential analysis identified model-induced genes and treatment-reversed genes in each tissue (criteria in Materials and Methods). A Venn comparison of these reversal sets revealed substantial overlap between bone and the hippocampus, indicating a shared TFDF-responsive program (Fig. 3A). Among the top terms, the functional enrichment of reversal genes prioritized FOXO signaling (Fig. 3B). In defining treatment-reversed genes, we considered both Model up, TFDF H down and Model down, TFDF H up patterns; to identify a robust cross-tissue pathology-related node, we then applied a stringent filter of Model up and TFDF H down in both tissues, which converged on DEPP1 as the only common transcript with concordant reversal (Fig. 3C). A complementary enrichment analysis of the same reversal gene sets revealed that FOXO signaling was a recurrent pathway in bone and the hippocampus (Fig. 3D). Gene set enrichment analysis (GSEA) further supported pathway directionality: FOXO-linked programs were negatively enriched in Model vs. Sham and shifted toward positive enrichment in TFDF-H vs. Model, which is consistent with TFDF-associated restoration of FOXO activity across tissues (Fig. 3E).

### TFDF down-regulates DEPP1 expression and restores autophagy–mitochondrial homeostasis across the hippocampus and bone

**Fig. 4.**
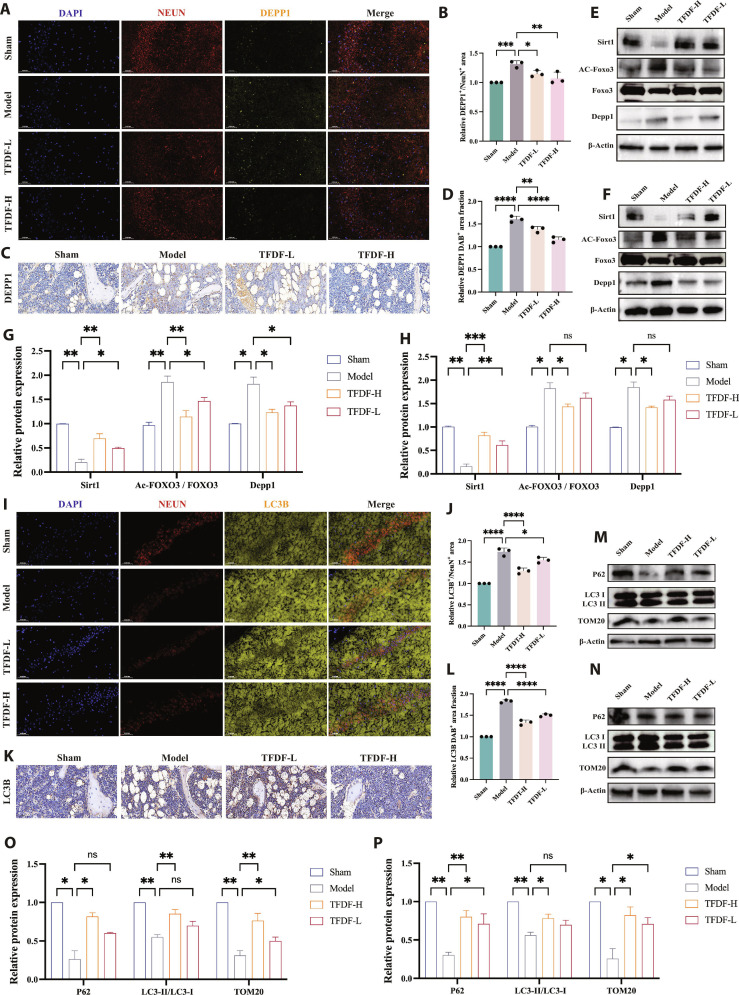
TFDF reduces DEPP1 expression and normalizes the expression of autophagy–mitochondrial markers in the hippocampus and bone of OVX–CUMS mice. (A) Representative hippocampal immunofluorescence images of NeuN (neurons, red) and DEPP1 (green) in the Sham, Model, TFDF-L, and TFDF-H groups; nuclei are stained with DAPI (blue). (B) Quantification of the DEPP1/NeuN double-positive area in the hippocampus. (C) Representative DEPP1 immunohistochemistry in trabecular bone. (D) Quantification of the DEPP1-positive area in bone. (E and F) Representative Western blots for SIRT1, FOXO3, and DEPP1 in the hippocampus (E) and bone (F). (G and H) Densitometric analysis of SIRT1, FOXO3, and DEPP1 expression in the hippocampus (G) and bone (H) normalized to that of β-actin. (I) Representative hippocampal immunofluorescence for NeuN (red) and LC3B (green). (J) Quantification of the hippocampal LC3B/NeuN double-positive area. (K) Representative LC3 immunohistochemistry in trabecular bone. (L) Quantification of the LC3-positive area in bone. (M and N) Western blots for p62, LC3B, and TOM20 in the hippocampus (M) and bone (N). (O and P) Densitometric analysis of p62, LC3B, and TOM20 expression in the hippocampus (O) and bone (P), normalized to that of β-actin.

At the tissue level, TFDF attenuated OVX–CUMS-induced pathway disturbances in both the hippocampus and bone and partly restored autophagy-related indices. In the hippocampus, double immunofluorescence (IF) staining for NeuN and DEPP1 showed a pronounced increase in neuronal DEPP1 signal in the Model group compared with the Sham group, whereas TFDF treatment reduced DEPP1 staining (Fig. 4A). Quantitative analysis confirmed a higher DEPP1/NeuN-positive area fraction in the Model group than in the Sham group, with significantly lower values in TFDF-treated mice (Fig. 4B and Fig. S1). In bone, DEPP1 immunohistochemistry revealed strong DEPP1 staining on trabecular surfaces in the Model group, which was diminished by TFDF (Fig. 4C), with quantitative image analysis showing a significant reduction in the DEPP1-positive area (Fig. 4D). Concordantly, hippocampal Western blots revealed decreased SIRT1 expression and increased DEPP1 and acetylated FOXO3 (Ac-FOXO3) expression in the Model group; TFDF increased SIRT1 expression and reduced DEPP1 and Ac-FOXO3 expression, while total FOXO3 protein expression was measured in parallel and is shown in the corresponding blots (Fig. 4E and F). Western blots from bone tissue revealed a recapitulation of the hippocampal pattern, with TFDF increasing SIRT1 expression and decreasing DEPP1 and AC-FOXO3 expression relative to those in the Model group (Fig. 4G), as supported by the densitometry results (Fig. 4H).

Autophagy readouts were tracked with these pathway corrections. In the hippocampus, NeuN/LC3B double IF indicated a reduced LC3B signal in Model animals that was restored by TFDF (Fig. 4I), with quantification confirming increased LC3B/NeuN-positive area fractions (Fig. 4J). In bone, LC3 immunohistochemistry increased in the TFDF group compared with that in the Model group (Fig. 4K), as shown by the corresponding quantification (Fig. 4L). Finally, Western blot analysis of autophagy/mitochondrial markers revealed that compared with the Model group, TFDF increased LC3-II (and/or the LC3-II/I ratio), decreased p62/SQSTM1, and restored TOM20 expression in the hippocampus and bone (Fig. 4M and N), as shown by the densitometry results (Fig. 4O and P).

### TFDF restores SIRT1–FOXO3–DEPP1 signaling and the autophagy–mitochondrial balance in MC3T3-E1 cells

**Fig. 5.**
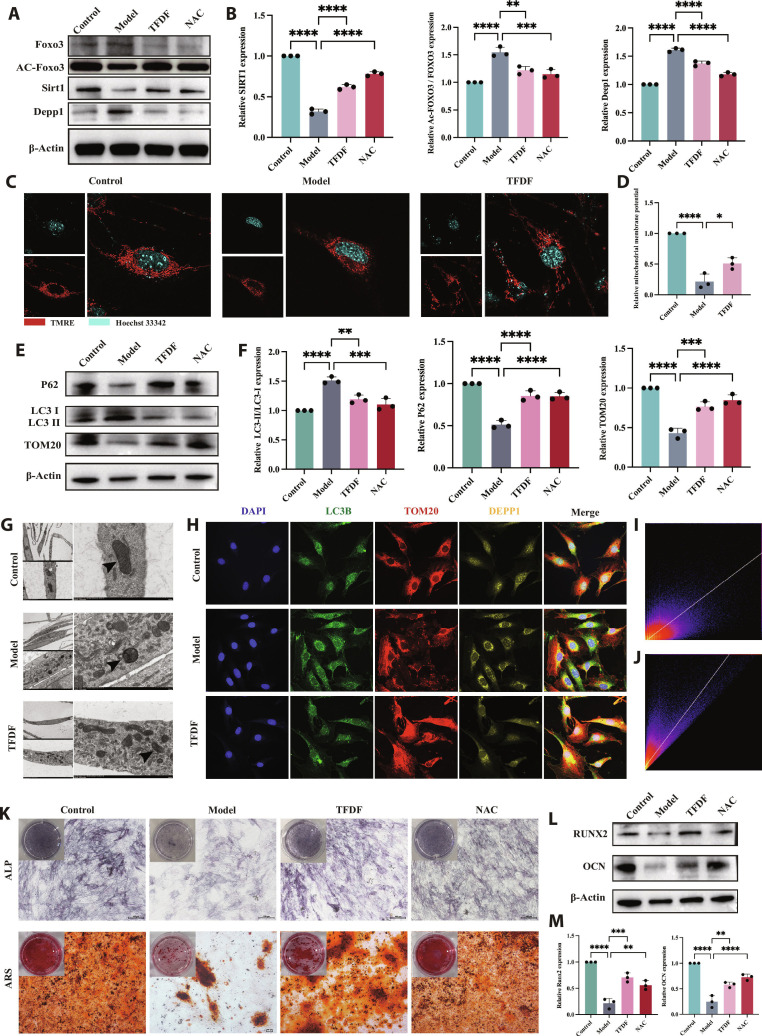
TFDF normalizes the SIRT1–FOXO3–DEPP1 axis, rebalances excessive autophagy, and rescues mitochondrial and osteogenic functions in MC3T3-E1 cells. MC3T3-E1 cells were assigned to Control, Model (H_2_O_2_ injury), TFDF, or NAC (positive antioxidant control) groups. Immunoblotting revealed that SIRT1 down-regulation, FOXO3 hyperacetylation, and DEPP1 up-regulation after H_2_O_2_ were reversed by TFDF (A), as determined by densitometry (B). TMRE microscopy and quantification revealed ΔΨm loss in the Model group and restoration by TFDF (C and D). Autophagy/mitochondrial markers indicated over-autophagy under injury that were recalibrated toward baseline by TFDF (E and F). TEM revealed autophagosome accumulation and swollen mitochondria in the Model group, which were mitigated by TFDF (G). Multicolor IF (LC3B/TOM20/DEPP1) revealed increased LC3B puncta, TOM20 fragmentation, and DEPP1 elevation in the Model group, all of which improved with TFDF (H); colocalization analyses confirmed normalization of LC3B–TOM20 (mitophagy coupling) and a reduction in TOM20–DEPP1 coupling by TFDF (I and J). Osteogenic function assays demonstrated TFDF-mediated recovery of ALP activity and ARS mineral deposition (K), accompanied by increased RUNX2 expression and OCN/ALP expression, as determined by Western blotting (L and M). Data are presented as the mean ± SEM, with *n* indicated on the plots; the statistical tests and multiple-comparison procedures are described in Materials and Methods. Abbreviations: TFDF, total flavonoids of *Drynaria fortunei*; NAC, N-acetyl-L-cysteine; TMRE, tetramethylrhodamine ethyl ester; LC3, microtubule-associated protein 1 light chain 3; OCN, osteocalcin.

In H_2_O_2_-treated MC3T3-E1 cultures, the pathway profile was consistent with a stressed state: SIRT1 protein was reduced, acetyl-FOXO3 was increased (with total FOXO3 relatively unchanged), and DEPP1 was elevated; TFDF partly normalized these changes, whereas N-acetyl-L-cysteine (NAC) produced qualitatively similar but generally weaker effects (Fig. 5A and B). H_2_O_2_ markedly reduced mitochondrial membrane potential (ΔΨm), as indicated by decreased tetramethylrhodamine ethyl ester, perchlorate (TMRE) fluorescence, and TFDF significantly attenuated this loss of ΔΨm (Fig. 5C and D). Autophagy markers indicated a dysregulated, overactivated autophagy state with impaired mitochondrial quality control in the Model group, characterized by LC3-II accumulation together with reduced p62 and decreased TOM20 expression. TFDF shifted these markers toward control levels (moderating LC3-II, increasing p62, and elevating TOM20), whereas NAC produced a partial correction (Fig. 5E and F). Ultrastructurally, transmission electron microscopy revealed numerous double-membrane autophagosomes and swollen mitochondria with reduced and disorganized cristae after H_2_O_2_ treatment, whereas TFDF-treated cells showed fewer autophagosomes and better preserved mitochondrial morphology (Fig. 5G). Four-color IF showed abundant LC3B puncta, fragmented TOM20-positive mitochondrial networks, and increased DEPP1 signal in the Model group; TFDF treatment reduced LC3B puncta, partially restored TOM20 network continuity, and decreased DEPP1 immunoreactivity (Fig. 5H). Quantitative colocalization analyses indicated enhanced mitophagy under oxidative injury, with increased LC3B–TOM20 overlap and elevated TOM20–DEPP1 colocalization in the Model group; both indices were significantly reduced by TFDF, shifting toward control values (Fig. 5I and J). Functionally, TFDF improved osteoblastic performance: ALP staining and Alizarin Red S (ARS) mineralization, both suppressed by H_2_O_2_, were increased in TFDF-treated cultures compared with the Model group (Fig. 5K). Consistent with these findings, Western blotting showed higher levels of RUNX2 and osteogenic markers in TFDF-treated cells than in H_2_O_2_-exposed Model cells (Fig. 5L and M).

### TFDF restores SIRT1–FOXO3–DEPP1 signaling and the autophagy–mitochondrial balance in HT22 cells

**Fig. 6.**
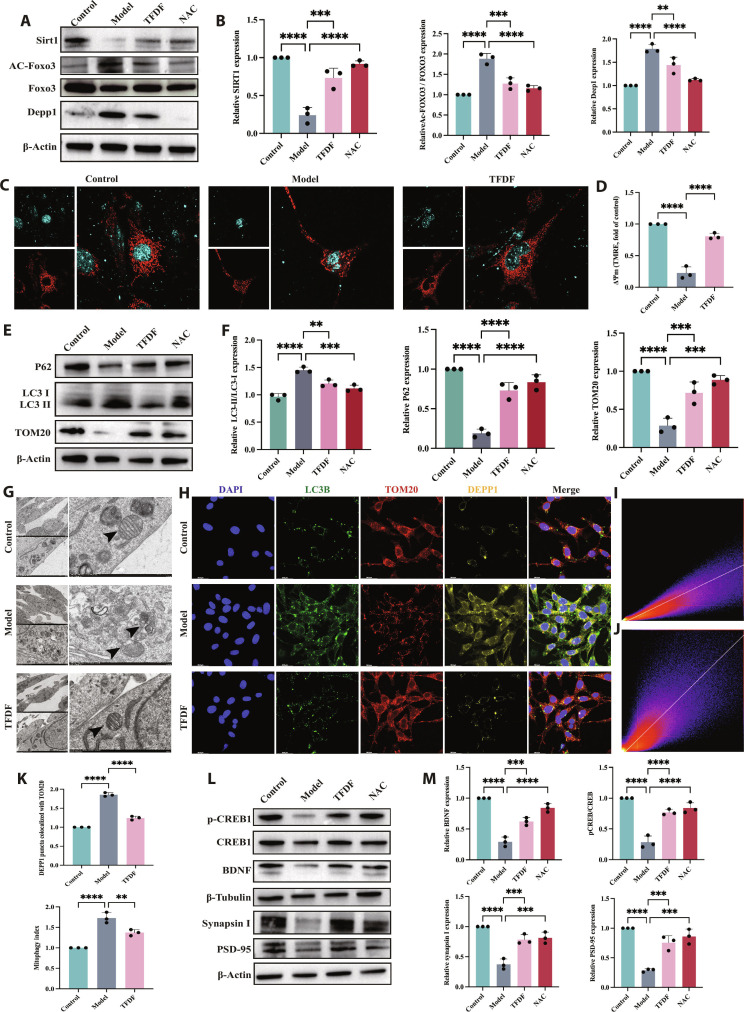
TFDF mitigates oxidative injury in HT22 cells by restoring SIRT1–FOXO3–DEPP1 signaling and autophagy–mitochondrial homeostasis, thereby improving neuroplasticity. Cells were assigned to Control, Model (H_2_O_2_), TFDF, or NAC (antioxidant control) groups. Western blots showing SIRT1–FOXO3–DEPP1↑ after injury and reversal by TFDF (A and B). TMRE imaging revealed ΔΨm loss in the Model group and rescue by TFDF (C and D). Autophagy/mitochondrial markers were recalibrated toward baseline by TFDF (E and F), which is consistent with TEM showing fewer autophagosomes and preserved cristae (G). IF (DAPI/LC3B/TOM20/DEPP1) demonstrated reduced LC3B puncta, increased TOM20 integrity, and decreased DEPP1 with TFDF (H), which was supported by colocalization readouts (DEPP1–TOM20 and LC3B–TOM20) (I and J). Quantification of DEPP1–TOM20 colocalization, and the mitophagy index is shown in (K). TFDF further increased the expression of BDNF and p-CREB/CREB and restored the expression of synapsin I and PSD-95 (L and M). Data are presented as the mean ± SEM; statistics and replicate numbers are provided in Materials and Methods. Abbreviations: TFDF, total flavonoids of *Drynaria fortunei*; NAC, N-acetyl-L-cysteine; TMRE, tetramethylrhodamine ethyl ester.

In H_2_O_2_-injured HT22 cells, the SIRT1–FOXO3–DEPP1 profile was consistent with that in the stressed state: the level of SIRT1 decreased, the level of acetylated FOXO3 (Ac FOXO3) increased, with only a modest change in total FOXO3, and the level of DEPP1 increased; TFDF (CCK-8-guided dose) reversed these alterations, whereas NAC resulted in a similar but generally weaker correlation (Fig. 6A and B). The mitochondrial membrane potential collapsed after injury, as indicated by the loss of the TMRE signal, and TFDF significantly restored the signal (Fig. 6C and D). Autophagy and mitochondrial markers were dysregulated and overactivated with organelle injury in the Model group, with LC3B accumulation, p62 depletion, and TOM20 loss; TFDF restored these markers to baseline levels, and NAC partly normalized them (Fig. 6E and F). Ultrastructurally, numerous double-membrane autophagosomes and swollen mitochondria appeared in the Model group but were reduced by TFDF (Fig. 6G). Multicolor IF revealed abundant LC3B puncta, fragmented TOM20, and high DEPP1 under injury; TFDF decreased the number of LC3B puncta, reestablished TOM20 continuity, and decreased DEPP1 expression (Fig. 6H). Colocalization analyses supported these changes: DEPP1–TOM20 coupling was elevated by injury and reduced by TFDF, whereas the LC3B–TOM20 mitophagy index was high in the Model group and normalized by TFDF (Fig. 6I and K). Finally, the expression levels of the following neuronal plasticity markers improved: relative to the Model group, TFDF increased BDNF and p-CREB/CREB expression and restored synapsin I and PSD 95 expression (Fig. 6L and M).

### DEPP1 bidirectionally modulates autophagy–mitochondrial stress in MC3T3-E1 and HT22 cells

**Fig. 7.**
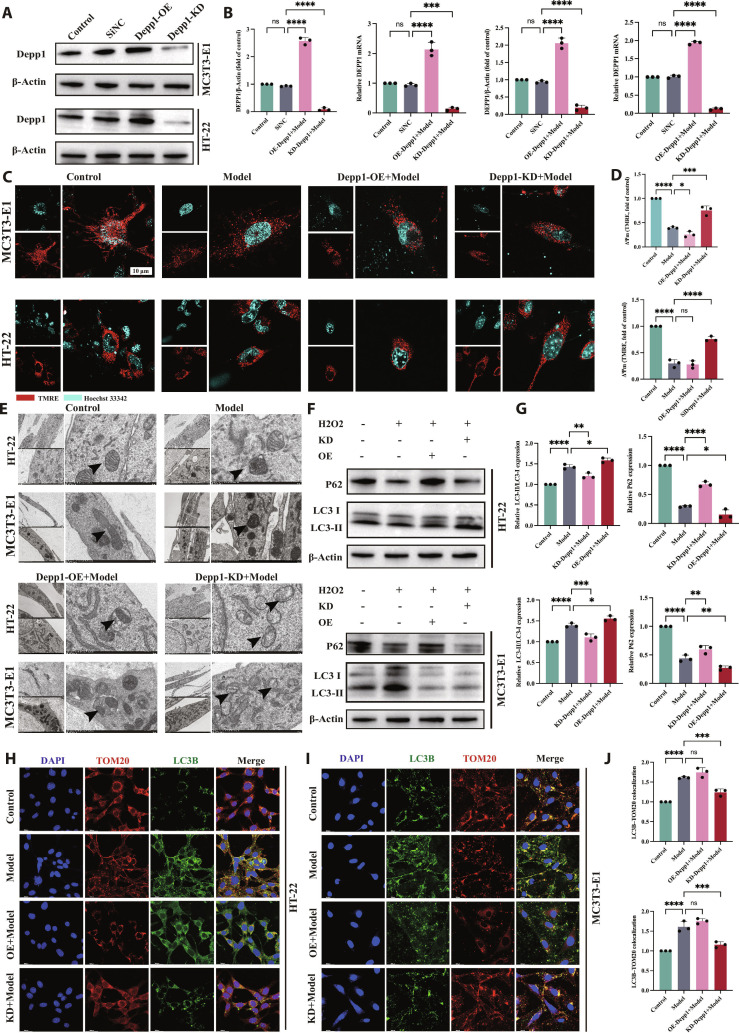
DEPP1 bidirectionally alters autophagy–mitochondrial coupling under oxidative injury in HT22 and MC3T3-E1 cells. DEPP1 expression was reduced by siRNA (KD) or increased by plasmid (OE); cells were exposed to H_2_O_2_ to model injury. Protein and mRNA assays confirmed effective KD/OE in both lines (A and B). TMRE imaging revealed ΔΨm loss in the Model group, partial recovery in the KD+Model group, and a further decrease in the OE+Model group (C and D). TEM revealed swollen mitochondria and autophagosomes in the Model group, fewer autophagosomes after KD, and abundant autophagosomes after OE (E). Western blots demonstrated model-associated LC3-II accumulation, p62 depletion, and TOM20 reduction; KD shifted these toward control, whereas OE intensified them (F) with densitometry in (G). LC3B/TOM20 immunofluorescence revealed parallel changes in puncta burden and mitochondrial network integrity, as shown by the statistical data in (J) (H to J). Group labels: Control, Model, KD+Model, and OE+Model. Data are presented as the mean ± SEM; replicate numbers and statistics are provided in Materials and Methods.

To define the role of DEPP1 in injury-induced autophagy and mitochondrial stress, we manipulated Depp1 by siRNA knockdown (KD) or plasmid overexpression (OE) and challenged MC3T3-E1 and HT22 cells with H_2_O_2_. Successful bidirectional modulation was verified at the protein and mRNA levels in both cell types (Fig. 7A and B). The mitochondrial membrane potential, assessed by TMRE, decreased in the injury model relative to that in the control, was partially restored by KD plus injury, and decreased further with OE plus injury in both lines (Fig. 7C and D). Transmission electron microscopy corroborated these functional results: the Model group displayed swollen mitochondria with disrupted cristae and frequent double-membrane autophagosomes; fewer autophagosomes but residual mitochondrial injury were observed in the KD plus injury group, whereas abundant autophagosomes and mitophagic vacuoles were observed in the OE plus injury group (Fig. 7E). Consistently, Western blots of autophagy markers indicated that autophagy was dysregulated and overactivated in the Model group that was tempered by KD plus injury and exacerbated by OE plus injury across both cell types (Fig. 7F and G). IF staining for LC3B and TOM20 revealed the same pattern: cells in the Model group displayed dense LC3B puncta and fragmented TOM20 networks; KD plus injury reduced puncta and improved TOM20 continuity, whereas OE plus injury further increased puncta and fragmentation, with quantification confirming these trends (Fig. 7H and J). Together, these data support DEPP1 as a bidirectional regulator of the autophagy–mitochondrial phenotype under oxidative stress.

### DEPP1 KD does not attenuate TFDF protection in MC3T3-E1 and HT22 cells

**Fig. 8.**
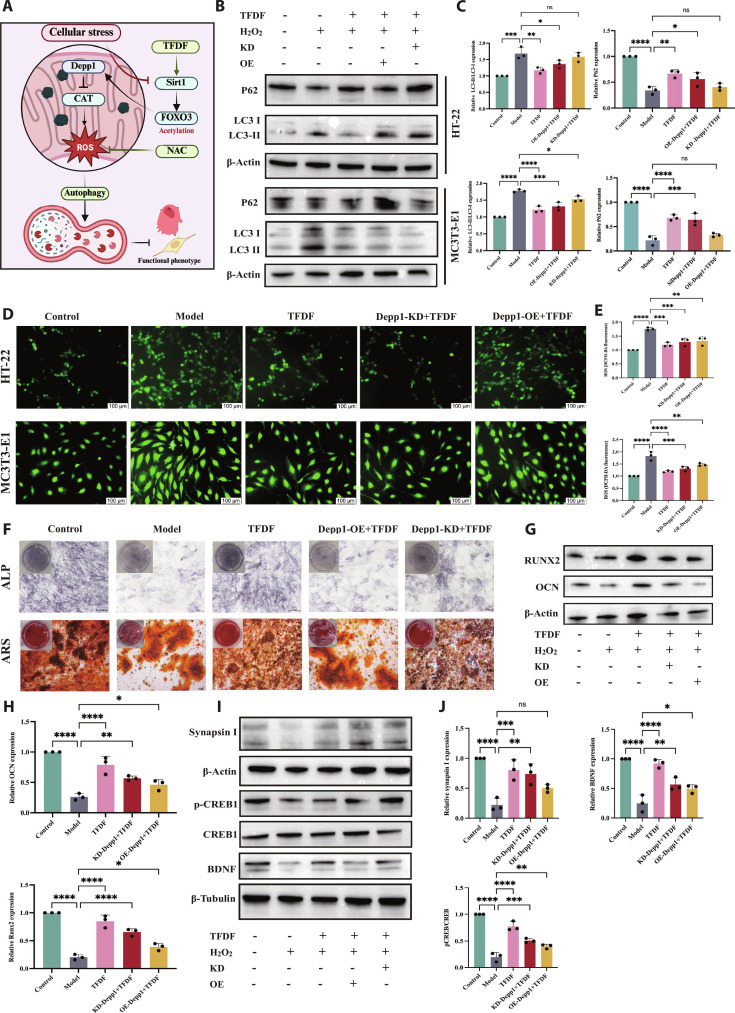
DEPP1 knockdown with TFDF maintains and often augments pathway activity and functional rescue in injured osteoblasts and neurons. (A) Schematic of the gene–drug design and hypothesized placement of DEPP1 downstream of TFDF-responsive signaling. (B) Autophagy/mitochondrial Western blots (LC3-I/II, p62, and TOM20) in MC3T3-E1 and HT22 cells under Control, Model, siDEPP1+Model, TFDF+Model, and siDEPP1+TFDF+Model conditions. (C) Densitometry for panel (B). (D) Representative ROS fluorescence micrographs. (E) Quantification of intracellular ROS levels. (F) Osteogenic function of MC3T3-E1 cells: ALP staining (day 7) and ARS mineralization (days 14 to 21). (G) Osteogenic markers (RUNX2, OCN, and ALP) were measured by Western blotting. (H) Corresponding quantification. (I) Neuronal plasticity markers in HT22 cells (BDNF, p-CREB/CREB, Synapsin I, and PSD-95) were measured by Western blotting. (J) Quantification. In both cell types, siDEPP1 and TFDF each improved the injury phenotype, and siDEPP1+TFDF achieved comparable or greater improvement without occluding the effects of TFDF. Statistical tests and *n* values are provided in Materials and Methods and on the plots.

Under oxidative injury, DEPP1 KD (siRNA-mediated) and TFDF treatment corrected the autophagy–mitochondrial signature in MC3T3-E1 and HT22 cells, and the KD plus TFDF combination resulted in equal or greater corrections across readouts. Mechanistically (Fig. 8A), the Model group revealed a dysregulated, overactivated autophagy phenotype with LC3 II accumulation, p62 depletion, and TOM20 loss; KD partially normalized these markers, and TFDF alone produced a similar change. In both cell types, compared with the Model group treatment, the KD plus TFDF treatment further decreased LC3 II expression, restored p62 expression, and increased TOM20 expression (Fig. 8B and C). Cellular stress followed the same pattern: reactive oxygen species (ROS) increased with injury and were reduced by either KD or TFDF, with the combination yielding the lowest ROS levels (Fig. 8D and E). Lineage-specific functions improved concordantly. In osteoblasts, ALP activity and ARS mineralization, both of which were suppressed by injury, increased in response to KD or TFDF and were greatest in response to KD plus TFDF (Fig. 8F), which was accompanied by increased levels of RUNX2, OCN, and ALP expression, as determined by Western blotting (Fig. 8G and H). In neurons, BDNF, p-CREB/CREB, synapsin I, and PSD 95 increased with KD or TFDF and were further improved by KD plus TFDF (Fig. 8I and J). Across endpoints, DEPP1 KD did not diminish TFDF benefits; several measures showed additive gains versus either intervention alone. These gene–drug interactions are most compatible with a model in which DEPP1 functions as a downstream stress–autophagy mediator within a broader SIRT1-centered network; TFDF can also act through DEPP1-independent or parallel pathways rather than relying on intact DEPP1 as its primary point of action.

### Docking and functional perturbation support SIRT1 as a key TFDF-dependent effector in osteoblasts and neurons

**Fig. 9.**
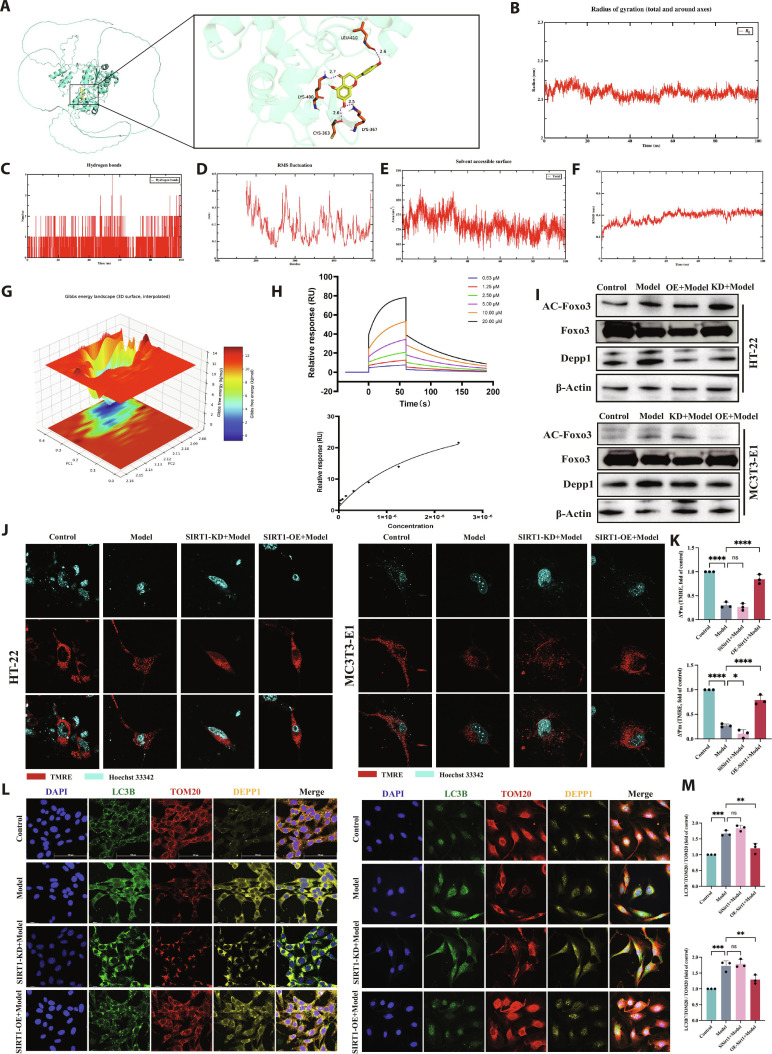
Docking and functional perturbation support SIRT1 as a key TFDF-responsive effector. (A) Docked pose of naringenin in the SIRT1 activator pocket with key hydrogen-bond and hydrophobic contacts indicated. (B) Radius of gyration, (C) number of protein–ligand hydrogen bonds, (D) backbone RMSF, (E) solvent-accessible surface area, and (F) ligand RMSD, all indicating rapid stabilization after ~10 ns and maintenance of a compact, well-behaved complex. (G) Free-energy landscape of the SIRT1–naringenin trajectory plotted along PC1 and PC2 (Δ*G* = −*k*_B_*T* ln *P*), showing a dominant low-energy basin corresponding to the bound state. (H) SPR analysis of SIRT1–naringenin binding, showing concentration-dependent sensorgrams and a 1:1 Langmuir fit consistent with specific interaction. (I) Western blots of FOXO3 and Depp1 (HT22 and MC3T3-E1) after Sirt1 knockdown (KD) or overexpression (OE). (J) TMRE staining (ΔΨm) and (K) corresponding quantification: ΔΨm decreases with KD and increases with OE. (L) Triplex IF (LC3, TOM20, and DEPP1) showing the autophagy burden, mitochondrial network integrity, and DEPP1 levels across KD/OE conditions. (M) Quantifications of puncta burden, TOM20 continuity, and DEPP1 intensity. Docking to DEPP1 with neoeriocitrin, naringin, and naringenin produced low-affinity, nonconvergent poses (not shown); docking and molecular dynamics results for naringin, naringenin, and the positive control are provided in the Supplementary Materials. Abbreviations: *R*_g_, radius of gyration; RMSF, root mean square fluctuation; SASA, solvent-accessible surface area; RMSD, root mean square deviation.

UPLC–Q-Orbitrap HRMS profiling of TFDF powder confirmed a flavonoid-rich composition dominated by naringin, naringenin chalcone, naringenin, eriodictyol, and kaempferol glycosides (including astragalin and kaempferol-7-O-glucoside), together with phenolic acids, procyanidin oligomers, and the dicarboxylic acid azelaic acid (Fig. S2). In serum from OVX–CUMS mice treated with TFDF, the same analytical workflow revealed a complex endogenous metabolite background overlaid with a subset of TFDF-derived small molecules, among which azelaic acid and several long-chain fatty-acid-related species showed the most robust and reproducible signals (Fig. S3). Integrating these compositional and exposure data with published pharmacokinetic and antioxidant profiles, we prioritized 3 representative flavonoids—naringin, naringenin, and neoeriocitrin—as candidate bioactive constituents for subsequent target-engagement analyses with SIRT1. Docking of these compounds to DEPP1 yielded low-affinity scores with diffuse, nonconvergent poses, whereas docking to SIRT1 identified a particularly favorable binding mode for naringenin, with neoeriocitrin also occupying the reported activator pocket (Table S3 and Fig. S4). Molecular dynamics simulations of the SIRT1–naringenin complex demonstrated a stable interaction, as reflected by a plateauing radius of gyration, stable hydrogen-bonding pattern, low RMSF values outside the flexible N-terminus, reduced SASA, and an equilibrated ligand root mean square deviation (RMSD), together with a dominant low-energy basin on the free-energy landscape (Fig. 9A to G). MM/GBSA energy decomposition over 0 to 100 ns yielded an average binding free energy of approximately −23.9 kcal·mol^−1^, with PRO411, PRO401, and VAL404 making major contributions to ligand stabilization. Consistent with these in silico data, surface plasmon resonance (SPR) experiments using SIRT1 immobilized on a CM5 chip showed that naringenin bound SIRT1 with clear, concentration-dependent sensorgrams that fitted a 1:1 Langmuir model, giving an equilibrium dissociation constant (*K*_D_) of 2.82 × 10^−6^ M (Fig. 9H). Together, these results identify SIRT1 as a direct target of naringenin and support the SIRT1–naringenin pair as a key TFDF-responsive effector module in this pathway. The in silico and biophysical results for naringin, neoeriocitrin, and the positive control are provided in the Supplementary Materials (Figs. S5 to S7).

Drug-independent perturbation of SIRT1 confirmed its functional position in this pathway. Under oxidative challenge, SIRT1 KD in HT22 and MC3T3-E1 cells reduced FOXO3 and increased acetylated FOXO3 (Ac-FOXO3) and DEPP1, whereas SIRT1 OE produced the opposite pattern (Fig. 9I and Fig. S8). Consistently, the mitochondrial membrane potential decreased with SIRT1 KD but increased with SIRT1 OE, as assessed by TMRE (Fig. 9J and K). Multicolor IF further showed that SIRT1 KD was associated with abundant LC3B puncta, fragmented TOM20 networks, and increased DEPP1 signal, whereas SIRT1 OE reduced LC3B puncta, preserved TOM20 continuity, and lowered DEPP1 (Fig. 9L and M). Together, these findings identify SIRT1 as a structurally compatible and biophysically validated binding site for at least one major TFDF flavonoid (naringenin) and, in conjunction with the comparative DEPP1 docking data, support SIRT1 as the principal TFDF-responsive regulator of the FOXO3–DEPP1–autophagy–mitochondria axis in osteoblasts and neurons.

### SIRT1 is required for full TFDF efficacy, and its OE potentiates pathway and organelle rescue

**Fig. 10.**
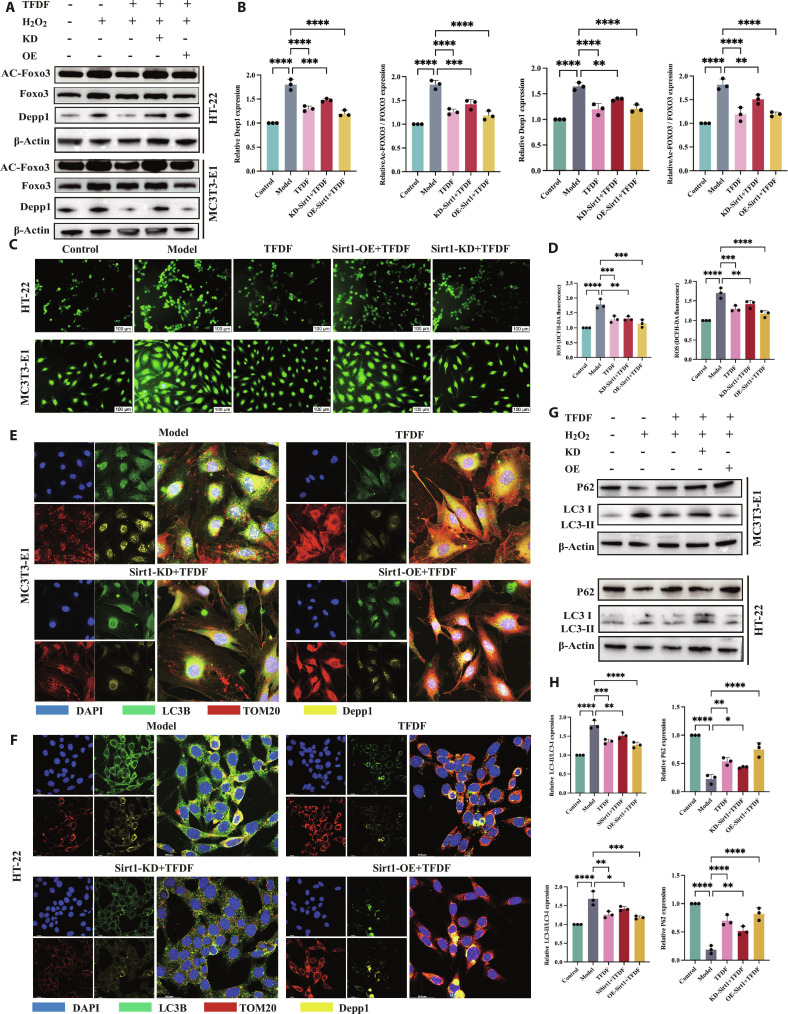
SIRT1 determines cellular responsiveness to TFDF in neurons and osteoblasts. Under H_2_O_2_ injury, cells were treated with TFDF alone or in combination with Sirt1 knockdown (KD) or overexpression (OE). Western blots showed that TFDF decreased acetyl-FOXO3 and DEPP1 levels and that these effects were abrogated by KD and strengthened by OE in HT22 and MC3T3-E1 cells (A, densitometry in B). ROS imaging/quantification demonstrated that injury-induced oxidative stress was reduced by TFDF, partially reversed by KD, and further reduced by OE (C and D). Immunofluorescence staining for LC3B/TOM20/DEPP1 revealed a TFDF-driven improvement in autophagy–mitochondrial morphology that was attenuated by KD and potentiated by OE in both cell types (E and F). Autophagy immunoblots (LC3-II and p62) confirmed the same interaction pattern (G, densitometry in H).

To test SIRT1 dependence, we combined TFDF with Sirt1 loss or gain of function under oxidative injury in HT22 and MC3T3-E1 cells. In both lines, compared with the control treatment, TFDF alone decreased the levels of Ac FOXO3 and DEPP1, whereas SIRT1 KD plus TFDF largely maintained high levels of Ac FOXO3 and DEPP1, and SIRT1 OE plus TFDF further reduced both readouts (Fig. 10A and B). Consistently, cellular ROS increased with injury, decreased in response to TFDF, remained elevated in response to KD plus TFDF, and decreased further in response to OE plus TFDF (Fig. 10C and D). Triple IF for LC3B, TOM20, and DEPP1 revealed that TFDF improved the phenotype in the Model group, with fewer LC3B puncta, more continuous TOM20, and lower DEPP1; these changes were attenuated by SIRT1 KD and augmented by SIRT1 OE across both cell types (Fig. 10E and F). Autophagy and mitochondrial markers mirrored the imaging data: the Model group displayed LC3 II accumulation with p62 depletion and decreased TOM20; TFDF shifted these indices toward the control, KD plus TFDF blunted this normalization, and OE plus TFDF enhanced it (Fig. 10G and H). These results indicate that SIRT1 is necessary for the full cytoprotective effect of TFDF and that increasing SIRT1 levels potentiates TFDF-mediated correction of FOXO3 acetylation, DEPP1, autophagy, and mitochondrial function in both osteoblasts and neurons.

## Discussion

**Fig. 11.**
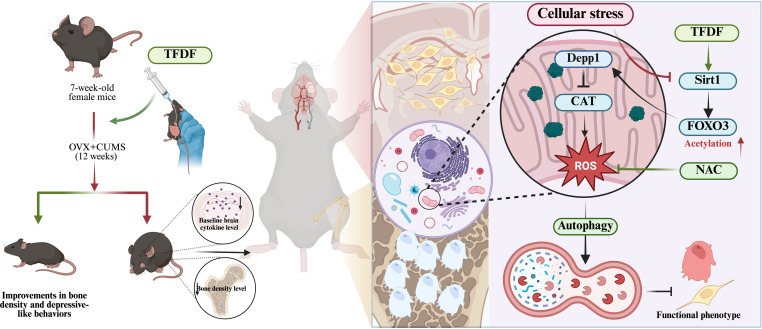
TFDF alleviates OVX–CUMS-associated bone–brain comorbidity by engaging SIRT1-centered stress–autophagy signaling. Left: Experimental framework: 7-week-old female mice underwent ovariectomy combined with chronic unpredictable mild stress (OVX+CUMS, 12 weeks) and received total flavonoids of *Drynaria fortunei* (TFDF), resulting in improved bone density and depression-like behaviors. Right: Working model: OVX+CUMS-related cellular stress elevates reactive oxygen species (ROS) and disrupts autophagy–mitochondrial homeostasis. TFDF activates SIRT1, reduces FOXO3 acetylation, suppresses stress-responsive DEPP1, and restores antioxidant capacity (e.g., catalase [CAT]), thereby lowering ROS (with N-acetyl-L-cysteine [NAC], shown as an antioxidant control) and rebalancing autophagy to support organelle quality control. These coordinated effects ultimately improve cellular function and the observed functional phenotype in bone- and brain-relevant cells.

In an OVX–CUMS mouse model, TFDF exerted coordinated benefits on both bone and brain: TFDF restored trabecular microarchitecture and bone turnover indices, ameliorated depressive-like behaviors, and preserved hippocampal neuronal morphology. Cross-tissue RNA-seq revealed a shared stress–autophagy signature in bone and hippocampus that was reversed by TFDF, with DEPP1 emerging as a convergent transcript in both tissues. Consistent with this, tissue and cellular assays demonstrated a coherent correction along the SIRT1–FOXO3–DEPP1–autophagy–mitochondria axis: TFDF partially restored SIRT1 and FOXO3, reduced DEPP1, normalized autophagic flux, improved mitochondrial integrity, and rescued osteogenic and neuroplastic markers. Gene–drug interaction experiments further refined the pathway hierarchy. DEPP1 OE attenuated the protective actions of TFDF, whereas DEPP1 KD did not abolish TFDF efficacy, indicating that DEPP1 functions primarily as a downstream stress–autophagy mediator rather than the initial pharmacologic target. In contrast, molecular docking, molecular dynamics simulations, and functional perturbation experiments collectively supported SIRT1 as a stable binding partner for major TFDF flavonoids, and SIRT1 KD blunted, while SIRT1 OE enhanced, TFDF responses, thereby positioning SIRT1 as the central TFDF-responsive regulator orchestrating FOXO3 acetylation, DEPP1 expression, autophagy, and mitochondrial homeostasis in osteoblasts and neurons (Fig. 11).

Emerging evidence indicates that OP and depression share several pathophysiological mechanisms, including chronic low-grade inflammation, oxidative stress, dysregulated (often excessive) autophagy with impaired flux, hypothalamic–pituitary–adrenal (HPA) axis hyperactivity, and mitochondrial dysfunction, that collectively drive parallel neural and skeletal degeneration [27–29]. Such convergent derangements reinforce the concept of an interorgan “brain–bone axis”, wherein central nervous system and bone pathologies can influence each other’s vulnerability and outcomes through endocrine, immune, and neural pathways [30]. For example, proinflammatory cytokines (e.g., tumor necrosis factor-α and interleukin-6) implicated in neuroinflammation and depressive pathology can simultaneously promote osteoclast-driven bone resorption and fragility; likewise, chronic HPA axis hyperactivation with excess glucocorticoids is detrimental to both hippocampal neurons and bone-forming osteoblasts [31,32]. Furthermore, oxidative damage and mitochondrial deficits have been observed in both depressed neural tissue and osteoporotic bone, linking failures in cellular energy metabolism to neurodegeneration and impaired bone formation [33,34]. In this context, our data do not directly demonstrate bone–brain communication per se but instead reveal a shared SIRT1–FOXO3–DEPP1-mediated stress–autophagy module that is simultaneously engaged in bone and the hippocampus during comorbidity and can be corrected by TFDF.

Recent pharmacological insights into TFDF and its key constituents provide mechanistic support for such dual neuroprotective and osteoprotective effects [35–37]. Naringin, a major Drynaria flavonoid, has been identified as a potent autophagy activator: it enhances autophagic flux via AMPK–SIRT1 signaling, which in turn deacetylates FOXO3 and up-regulates cytoprotective genes [38–40]. By increasing SIRT1–FOXO3 activity, naringin helps maintain mitochondrial homeostasis and reduces oxidative stress and inflammation in various models, indicating a conserved mechanism of cellular protection relevant to both bone and neural tissue [38,41]. Neoeriocitrin, another pivotal TFDF component, was recently shown to bind and stabilize Beclin1, a core autophagy initiator, thereby preventing its degradation and increasing autophagy in osteogenic stem cells; this translated into enhanced osteoblast differentiation and bone regeneration in vivo [42]. In parallel, the flavonoids of TFDF engage in neurotrophic signaling; for instance, the activation of CREB by naringin has been shown to restore hippocampal neurogenesis and reverse chronic stress-induced depressive behaviors [19,43]. Our findings extend this work by linking these known actions to a common SIRT1–FOXO3–DEPP1 axis in both osteoblasts and hippocampal neurons, integrating autophagy–mitochondrial quality control with lineage-specific readouts of bone formation and synaptic plasticity.

In addition, considering TFDF in the broader landscape of SIRT1 activators and autophagy modulators is informative. Small-molecule SIRT1 activators such as resveratrol and synthetic SRT compounds, as well as autophagy-targeting agents such as rapamycin and metformin, have shown beneficial effects in models of OP, depression, neurodegeneration, and metabolic syndrome [44–46] However, they are typically evaluated in single-organ or single-disease settings, and their clinical use can be limited by off-target actions, systemic metabolic effects, or narrow therapeutic windows. In contrast, TFDF is a clinically used, standardized natural product preparation for OP with a relatively favorable safety record, and in the present comorbid OVX–CUMS model, it engaged the SIRT1–FOXO3–DEPP1 pathway in both bone and the hippocampus, thereby improving skeletal and behavioral endpoints within the same animals. Although head-to-head comparisons with other SIRT1 activators or autophagy modulators were not performed here, our results suggest that TFDF may offer a practical “single agent, dual organ” strategy for treating postmenopausal OP with depression, which merits further comparative evaluation.

### Innovation and limitations

This study has several methodological and conceptual strengths. First, to our knowledge, this is the first study to investigate TFDF in an integrated comorbidity model that combines ovariectomy and chronic unpredictable mild stress, allowing simultaneous assessment of skeletal and neural outcomes within one framework. Second, by performing cross-tissue RNA-seq and identifying DEPP1 as a shared stress–autophagy transcript in both bone and the hippocampus, followed by in vitro gain- and loss-of-function studies, we begin to map a unified SIRT1–FOXO3–DEPP1 signaling module across osteoblasts and neurons. Third, the demonstration that SIRT1 KD attenuates and that SIRT1 OE potentiates TFDF effects, together with the results of docking and molecular dynamics analyses, provides convergent support for a SIRT1-dependent mechanism of action.

Nonetheless, several limitations should be acknowledged. Mechanistically, our in vivo evidence remains correlative: although cellular perturbation experiments implicate SIRT1 as a key TFDF-dependent regulator and DEPP1 as a downstream mediator, direct genetic validation in vivo, for example, using conditional Sirt1 or Depp1 KD or OE in bone and hippocampal compartments, would more definitively prove pathway necessity. In addition, H_2_O_2_-based in vitro models capture the oxidative stress component of OVX–CUMS but not its full chronic, multifactorial milieu. Finally, although we discuss the brain–bone axis conceptually, our experiments do not directly track bone- or brain-derived signals in circulation and therefore cannot establish causal bone–brain communication. These issues will need to be addressed in future work.

### Outlook

Future work should more precisely define bone–brain communication in OP–depression comorbidity. Increasing evidence supports a bidirectional brain–bone axis mediated by endocrine, neural, and immune signals, and our identification of a shared SIRT1–FOXO3–DEPP1 stress–autophagy module offers a concrete entry point into this network. It will be important to clarify whether TFDF primarily reprograms osteoblasts, hippocampal neurons, or both, thereby modulating circulating osteokines and neuromodulators that couple skeletal and brain homeostasis. Longitudinal multi-omics profiling, together with cell type-specific genetic perturbation and targeted pharmacology, may help map these communication routes and nominate tractable targets for integrated treatment of osteoporotic depression.

In summary, TFDF provides a mechanism-based therapeutic option for OP–depression comorbidity in the OVX–CUMS setting. Our data support SIRT1 as a key TFDF-dependent effector and DEPP1 as a downstream stress–autophagy mediator within a shared SIRT1–FOXO3–DEPP1 axis, establishing an autophagy–mitochondrial homeostasis-based mechanistic framework linking bone and the hippocampus in this model. Importantly, this work highlights the promise of TCM-derived EDNM agents such as TFDF as single agents capable of addressing both skeletal degeneration and mood-related pathology. Future efforts should combine in vivo genetic manipulation and target engagement assays with studies of bone–brain communication to further validate this pathway and accelerate clinical translation.

## Materials and Methods

### Experimental animals

Female C57BL/6J mice (7 weeks of age at arrival) were obtained from the Qinglongshan Animal Experimental Center (Nanjing, China). All procedures were conducted in the Animal Experiment Center of the Nanjing University of Chinese Medicine under an approved protocol (IACUC No. 202312A020). Animals were housed in a specific-pathogen-free facility with controlled temperature (22 to 24 °C), humidity (45% to 65 %), and a 12-h light/12-h dark cycle, with standard chow and water available ad libitum. Mice were acclimated for 7 days before any intervention.

Animals were randomly assigned to experimental groups (Sham, OVX + CUMS model, TFDF-L, TFDF-H, and E2 positive control; group sizes are reported in figure legends). Prespecified exclusion criteria included surgical failure, severe illness unrelated to study procedures, or technical artifacts that precluded analysis; any exclusions are documented in the corresponding figure legends. All efforts were made to minimize animal number and suffering, and environmental enrichment was provided according to institutional guidelines.

### Reagents

TFDF was supplied as capsules by Beijing Qihuang Pharmaceutical Co., Ltd. (China; NMPA approval Z20030007; 0.25 g per capsule). TFDF is a state-approved, standardized total flavonoid preparation of *Drynaria fortunei*; according to the manufacturer’s quality specification, naringin and neoeriocitrin are used as marker compounds for batch release, and each production batch must meet predefined content limits. Immediately before dosing, the capsule shell was opened and the brown powder was weighed and suspended in purified water to the required working concentration. Mice received TFDF by oral gavage once daily, 6 days per week at 132.9 mg/kg/day (low dose) or 265.8 mg/kg/day (high dose). A dosing volume of 10 ml/kg was used, corresponding to 13.29 mg/ml for the low dose and 26.58 mg/ml for the high dose.

Estradiol valerate tablets (Bujiale; Bayer HealthCare Co., Ltd., Guangzhou, China; NMPA approval J20171038; 1 mg per tablet) were finely ground and suspended in purified water immediately before use. The working suspension was 0.036 mg/ml, delivered by oral gavage at 10 ml/kg to achieve 0.36 mg/kg/day. The same vehicle and volume were given to appropriate controls. All administrations were performed at consistent times of day.

### Chemical profiling of TFDF by UPLC–Q-Orbitrap HRMS

TFDF powder was chemically profiled by ultraperformance liquid chromatography coupled to Q Orbitrap high-resolution mass spectrometry (UPLC–Q Orbitrap HRMS) using a validated flavonoid focused method. Briefly, TFDF capsules were opened, the powder was extracted with aqueous methanol, and the supernatant was analyzed in both positive and negative electrospray modes on a UPLC–Q Orbitrap platform. Accurate mass, MS/MS fragmentation patterns, and retention times were matched against reference standards and databases to annotate major constituents.

### Ovariectomy

Bilateral OVX was performed under anesthesia with ready-to-use 2,2,2-tribromoethanol administered intraperitoneally, following institutional guidelines for depth of anesthesia. Mice were placed in prone position, the dorsal skin was aseptically prepared, and 2 small flank incisions were made to exteriorize the ovaries. The oviducts were ligated and both ovaries were removed. Muscle and skin were closed with absorbable sutures. Sham animals underwent identical procedures except that the ovaries were exteriorized and returned intact [47]. Postoperative care included thermal support and analgesia as per facility policy. Animals were monitored daily and allowed to recover before subsequent procedures.

### Chronic unpredictable mild stress

After recovery from OVX or sham surgery, mice entered a CUMS schedule designed to induce persistent depressive-like behavior [48,49]. Stressors were applied once or twice per day in a pseudorandom order to prevent habituation, with no stressor repeated on consecutive days. The panel included commonly used mild stressors such as wet bedding, cage tilt, light–dark cycle perturbation, brief food or water deprivation, restraint, cold exposure or cold swim of short duration, tail pinch of brief duration, and exposure to a novel odor. The regimen was maintained for multiple weeks according to the study timeline, and was run in parallel with the assigned treatments. Behavioral readouts (for example, sucrose preference and open-field measures) were collected at predefined time points to verify induction of the depressive-like phenotype.

### Preparation of mouse serum

After isoflurane anesthesia, blood was obtained from the ophthalmic vein into serum tubes, allowed to clot at room temperature for 30 min, and centrifuged at 2,500 rpm for 15 min. The supernatant was transferred to fresh tubes, flash frozen in liquid nitrogen, aliquoted, and stored at −80°C until analysis. Samples were collected at a consistent time of day within each experiment, handled on ice, and limited to a single freeze–thaw cycle. Grossly hemolyzed samples were excluded.

### Behavioral measurements

#### Tail suspension test

Mice were individually suspended by the tail with adhesive tape fixed to a horizontal bar inside a sound-attenuated chamber for 6 min. Immobility was defined as the absence of initiated movements other than those required for respiration or passive pendulum-like swinging. Sessions were video-recorded and scored offline by an investigator blinded to group assignment using time-stamped event logging. Trials were excluded a priori if a mouse detached from the hook, climbed onto its tail, or exhibited persistent escape-related behaviors that prevented reliable immobility scoring. The primary outcome was cumulative immobility time over 6 min [50].

#### Forced swim test

Animals were placed one at a time into a clear acrylic cylinder (inner diameter, 12 cm; height, 25 cm) filled with water at 24 ± 1 °C to a depth that prevented tail contact with the bottom and escape (≥15 cm). Behavior was recorded for 6 min with a side-mounted camera. Immobility was scored as the absence of active swimming or climbing, with only minimal movements to keep the head above water. Water was replaced between trials to avoid olfactory cues and temperature drift. Sessions were excluded if a mouse sank, climbed the wall, or floated motionless for the entire period. The primary endpoint was immobility time across 6 min [50].

#### Sucrose preference test

A 2-bottle choice procedure was used. Mice were habituated for 24 h with 2 bottles of water, followed by 24 h with 2 bottles of 1% (w/v) sucrose. Animals showing extreme baseline preference (<25% or >75%) during habituation were excluded. After 24-h water deprivation, testing was conducted for 10 h with one bottle of water and one bottle of 1% sucrose; bottle positions were swapped at 5 h to control for side bias. Bottles were preweighed, checked for leakage, and reweighed at the end of each interval; spillage controls were run in empty cages. Sucrose preference (%) was calculated as sucrose intake divided by total liquid intake [50,51].

#### Open field test

Locomotion and exploratory behavior were assessed in a square arena (40 × 40 × 40.5 cm) under dim illumination. Each mouse was placed in the center and allowed to explore for 10 min while a ceiling-mounted camera captured trajectory. Videos were analyzed in Any-maze (Stoelting, USA) by a blinded investigator. The primary metric was total distance traveled. Secondary indices (for example, time and entries in the center zone) were extracted when needed. The enclosure was cleaned with 70% ethanol between trials [50].

### Micro-CT analysis

Distal femora were scanned on a Skyscan 1276 system (Bruker, Belgium) at 70 kV, 200 μA, and 9 μm isotropic voxel size, with rotation step and frame averaging set according to manufacturer recommendations to minimize noise and ring artifacts. Reconstructions were performed with default beam-hardening correction; binarization thresholds were held constant across specimens. The trabecular region of interest (ROI) was defined in the distal metaphysis proximal to the growth plate and extended for a fixed number of slices to avoid partial-volume effects. CTAn v1.15.2.2 yielded BMD, BV/TV, bone surface-to-volume ratio (BS/TV), Tb.N, and Tb.Sp. Representative 2-dimensional sections and 3-dimensional renderings were generated with DataViewer v1.5.1.9 and CTvox v3.0.0 using identical visualization parameters for all groups.

### Hematoxylin and eosin staining

Femora were fixed in 4% paraformaldehyde for approximately 72 h, decalcified in 10% EDTA (pH 7.2 to 7.4) at room temperature for ~30 days with frequent solution changes, dehydrated through graded ethanol, and embedded in paraffin. Sections (4 μm) were deparaffinized in xylene, rehydrated, and stained with hematoxylin followed by eosin using a standardized timing sequence. Slides were cleared, mounted, and imaged under bright-field microscopy with uniform exposure settings. Morphology was evaluated in matched anatomical planes to ensure comparability.

### RNA-seq analysis

Hippocampus and femoral bone (~50 mg per sample) were homogenized, and total RNA was isolated with TRIzol. RNA integrity was verified (RIN threshold prespecified; only high-quality RNA advanced to library prep). Poly(A)+ libraries were prepared with the Fast RNA-seq Lib Prep Kit V2 and sequenced on the NovaSeq X Plus platform using paired-end chemistry. After adapter trimming and quality filtering, reads were aligned to the mouse reference genome with HISAT2 (v2.0.5). Gene-level counts were generated and normalized to FPKM to mitigate depth and length effects. Differential expression was performed with DESeq2 (v1.20.0) with Benjamini–Hochberg correction; significance required |log2 fold change| > 1 and adjusted *P* < 0.05. Hierarchical clustering used Euclidean distance on normalized expression values. Functional annotation employed clusterProfiler (v3.8.1) for Gene Ontology (GO) and Kyoto Encyclopedia of Genes and Genomes (KEGG); where indicated, GSEA was used to determine pathway directionality. All analyses were conducted with investigators blinded to group codes until statistics were finalized.

### Western blot

#### Sample preparation

Hippocampal and bone tissues, as well as MC3T3-E1 and HT-22 cells, were lysed on ice in radioimmunoprecipitation assay (RIPA) buffer supplemented with protease and phosphatase inhibitors. For acetyl-FOXO3 detection, lysates additionally contained deacetylase inhibitors (for example nicotinamide and TSA). Protein concentration was determined by bicinchoninic acid (BCA) assay. Equal amounts of protein (20 to 40 μg per lane) were resolved by sodium dodecyl sulfate–polyacrylamide gel electrophoresis (10% to 12% gels; 12% to 15% for LC3-I/II) and transferred to polyvinylidene difluoride (PVDF) membranes.

#### Blotting

Membranes were blocked in 5% bovine serum albumin (BSA; phospho/acetyl targets) or 5% nonfat milk (other targets) for 1 h at room temperature, incubated overnight at 4 °C with primary antibodies, washed, and then incubated with horseradish peroxidase (HRP)-conjugated secondary antibodies for 1 h at room temperature. Signals were developed by ECL and imaged under nonsaturating conditions. Densitometry was performed in ImageJ by investigators blinded to group allocation. Band intensities were normalized to β-actin or β-tubulin; for LC3, both LC3-II level and LC3-II/LC3-I ratio were reported; for p-CREB, the p-CREB/CREB ratio was used. For Western blot densitometry in animal tissues, band intensities were normalized to the mean value of the Sham group, which was set to 1.0.

#### Primary antibodies

Primary antibodies (supplier and catalog number are in parentheses) were as follows: DEPP1 (CUSABIO, CSB-PA865135LA01HU); Acetyl-FOXO3 (Lys271, #AF3771); SIRT1 (YT4302); LC3B (Immunoway, PT0235R); p62/SQSTM1 (CY5546); TOMM20 (CY5527); BDNF (Immunoway, PT0858R, YM8627); CREB-1 (Immunoway, PT0516R, YM8342); CREB-1 phospho-Ser133 (Immunoway, PT0863R, YM8632); PSD-95 (Immunoway, PT0455R, YM8292); Synapsin I (Immunoway, PT0602R, YM8411); RUNX2 (Servicebio, GB115631-100); OCN (Servicebio, GB11233-100); β-Actin (AB0011); and β-Tubulin (AB0039). Working dilutions followed manufacturer recommendations and were optimized empirically within a narrow range across experiments.

### Quantitative reverse-transcription polymerase chain reaction

Total RNA was extracted from hippocampus, femoral bone, and cultured cells using TRIzol or a column kit according to the manufacturer’s instructions, including on-column DNase treatment. RNA purity and concentration were assessed spectrophotometrically; samples with A260/280 between 1.8 and 2.1 were used. cDNA was synthesized from 500 to 1,000 ng of RNA using a reverse-transcription kit with random primers (Table S1). qPCR was performed with SYBR Green chemistry on a 96-well system with standard cycling and melt-curve analysis to verify single amplicons.

### Enzyme-linked immunosorbent assay

Serum concentrations of bone formation markers (bone-specific ALP and BGP or OCN), bone resorption markers (β-isomerized C-terminal telopeptide of type I collagen and tartrate-resistant acid phosphatase 5b), and endocrine stress mediators (norepinephrine and corticosterone) were measured by enzyme-linked immunosorbent assay (ELISA). For every ELISA, we generated a full standard curve supplied by the manufacturer and ran all study samples in duplicate; to check matrix effects, representative plasma was reassayed after a 1:2 dilution, and the resulting values remained proportional (linear regression *R*^2^ > 0.97), indicating negligible interference

### Immunohistochemistry

Paraffin-embedded femoral sections (≈4 μm) were dewaxed in xylene and rehydrated through graded ethanol to water. Heat-induced antigen retrieval was performed in citrate buffer (pH 6.0) at 95 °C for 10 to 30 min, followed by passive cooling to room temperature. Endogenous peroxidase was quenched with 3% H_2_O_2_ for 10 min. Sections were blocked with 5% BSA (or normal serum, as appropriate) for 30 min, then incubated overnight at 4 °C with primary antibodies DEPP1 (CUSABIO, CSB-PA865135LA01HU) and LC3 (Immunoway, PT0235R) at manufacturer-recommended dilutions. After 3 phosphate-buffered saline (PBS) washes (5 min each), HRP-conjugated secondary antibodies were applied for 30 min at room temperature. Signal was developed with DAB, nuclei were counterstained with hematoxylin, and slides were dehydrated, cleared, and mounted. Images were acquired under a bright-field microscope using identical exposure settings across groups.

### Immunofluorescence

IF was performed on mouse hippocampal sections and on MC3T3-E1 and HT22 cells using primary antibodies DEPP1 (CUSABIO, CSB-PA865135LA01HU), LC3 (Immunoway, PT0235R), and TOM20 (Immunoway, PT0287R). Brains were perfusion-fixed and cryosectioned; cells were fixed on coverslips. Samples were permeabilized and blocked, incubated with primary antibodies overnight at 4 °C, then with species-appropriate Alexa Fluor-conjugated secondary antibodies for 1 h at 37 °C, counterstained with 4′,6-diamidino-2-phenylindole (DAPI), and mounted in antifade medium. Images were acquired on a laser scanning confocal microscope (Olympus FV1000) under identical acquisition settings across groups. Quantification was performed in ImageJ/Imaris by blinded investigators, extracting mean fluorescence intensity and positive area for each marker, LC3 puncta per cell or NeuN-defined region, and colocalization indices (LC3–TOM20 and TOM20–DEPP1) using Pearson/Manders coefficients; 3 to 5 nonoverlapping fields per animal or coverslip were averaged for statistics.

### Serum TFDF constituents by UPLC-HRMS

Serum samples were collected from OVX–CUMS mice after TFDF or vehicle treatment and stored at −80 °C until analysis. For liquid chromatography–mass spectrometry (LC-MS), 100 μl of serum was mixed with 3 to 4 volumes of cold methanol, vortexed, and centrifuged, and the supernatant was injected into a UPLC-Q-Orbitrap high-resolution mass spectrometer. Chromatographic separation was performed on a C18 column using a water/acetonitrile gradient with 0.1% formic acid at a constant flow rate. Data were acquired in both positive and negative electrospray ionization modes over an appropriate *m*/*z* range and processed with vendor software to generate extracted ion chromatograms and MS/MS spectra. Candidate TFDF-derived constituents were assigned by matching accurate mass, isotope patterns, and fragment ions to TFDF extract profiles and reference databases, and by requiring absence (or near absence) in serum from untreated control animals.

### Cell culture and treatment

#### MC3T3-E1 osteoblastic cells

MC3T3-E1 (STCC20026P, Servicebio) were cultured in α-MEM (Gibco) supplemented with 10% fetal bovine serum (FBS) (Servicebio, G8003-100ML) and 1% penicillin–streptomycin at 37 °C in 5% CO₂ and saturated humidity. Cells were authenticated by the supplier and routinely tested mycoplasma-negative. Medium was refreshed every 2 to 3 days, and cells were passaged at 70% to 80% confluence using 0.25% trypsin–EDTA at a 1:3 ratio. Only passages 5 to 25 were used for experiments. Typical seeding densities were as follows: 6-well plates for Western blot, 2 to 3 × 10^5^ cells per well; 24-well glass coverslips for IF, 4 to 6 × 10^4^ cells per well; 96-well plates for imaging, 8 to 10 × 10^3^ cells per well.

#### HT-22 mouse hippocampal neuronal cells (HT-22 cell line)

HT-22 cells (STCC20011P, Servicebio) were maintained in RPMI-1640 (Gibco) with 10% FBS (Servicebio) and 1% penicillin–streptomycin under identical incubator conditions. Medium exchange, passaging, and quality control mirrored the MC3T3-E1 workflow. Seeding densities matched the assay type as above.

#### Concentration screening for TFDF and NAC

TFDF working concentrations for cell assays were determined by CCK-8 viability screening on graded concentrations in each cell line, selecting doses that preserved viability while providing protection against oxidative challenge. NAC served as an antioxidant comparator and was similarly screened to ensure the final vehicle content was ≤0.1% in all wells.

#### Oxidative injury model and treatment groups

For oxidative injury, MC3T3-E1 cells were exposed to 200 μM H_2_O_2_ for 4 h, and HT22 cells were exposed to 350 μM H_2_O_2_ for 24 h [52,53]. In our pilot Cell Counting Kit-8 (CCK-8) and TMRE assays, these conditions consistently produced an approximately 30% to 40% reduction in cell viability together with a marked loss of mitochondrial membrane potential, without causing gross detachment or necrosis, and are comparable to those used in previous oxidative stress models in MC3T3-E1 and HT22 cells. These parameters were chosen to model an acute oxidative stress episode superimposed on the chronic stress and estrogen deficiency present in the in vivo OVX–CUMS paradigm, allowing us to isolate the stress–autophagy–mitochondria component under controlled conditions.

#### Osteogenic induction and functional readouts in MC3T3-E1

For lineage function, MC3T3-E1 were switched to osteogenic induction medium (Procell, PD-033) according to the manufacturer’s protocol. ALP staining was performed on days 5 to 7 of induction and ARS mineralization on days 14 to 21. Signals were quantified by image analysis and ARS dye extraction, respectively. Parallel Western blots for RUNX2, ALP, and OCN were used to validate osteogenic status.

#### Stable KD of Sirt1 and Depp1

Stable gene interference used mouse kits Sirt1 (GeneID: 93759, Cat. RD307092) and Depp1 (GeneID: 213393, Cat. RD311303) following the manufacturers’ instructions. Briefly, cells were transduced at the indicated multiplicity of infection (MOI) in the presence of polybrene (4 to 8 μg/ml), incubated 24 to 48 h, and selected with puromycin at a concentration determined by a prior kill curve. KD efficiency was verified by qPCR (48 to 72 h after selection onset) and Western blot. Stable pools were expanded and used for assays within 3 passages postselection.

#### Transient OE of Sirt1 and Depp1

OE plasmids were Mm12441 for mouse Sirt1 (NM_019812.3, ORF 2214 bp; synonyms SIR2L1, Sir2, Sir2a, Sir2alpha) and Mm14340 for mouse Depp1 (NM_145980.2, ORF 618 bp; synonyms 8430408G22Rik, DeppFseg). Transfections used Lipo6000 (Beyotime, C0526-0.5 ml) per the supplier’s protocol. For 6-well plates, 2.0 to 2.5 μg of DNA/well with a 1:3 to 1:4 DNA:reagent ratio was used; complexes were formed for 15 to 20 min and added dropwise to antibiotic-free medium. Medium was refreshed after 6 h. Assays were performed 24 to 48 h posttransfection. Transfection efficiency and protein OE were confirmed by qPCR/Western blot before downstream analyses.

#### TMRE assay and nuclear counterstain

##### Mitochondrial membrane potential

Live MC3T3-E1 and HT-22 cells grown on glass coverslips were incubated with TMRE (GLPBIO, GC30053) in phenol-red-free medium (100 nM, optimized within 50 to 200 nM) for 20 min at 37 °C protected from light. Cells were rinsed once with warm medium and imaged immediately on a confocal microscope using identical acquisition settings across groups (Ex/Em ≈ 550/575 nm). In selected plates, a depolarization control was included by treating parallel wells with FCCP (10 μM, 10 min) before TMRE to verify dynamic range. For plate-reader assays (black 96-well), fluorescence was recorded at Ex/Em 549/575 nm and background-subtracted. ΔΨm was expressed as mean TMRE intensity per cell or per field and normalized to the FCCP control or vehicle as indicated.

##### Nuclear counterstain

After TMRE imaging (or in parallel wells), nuclei were labeled with Hoechst 33258, ready to use (Servicebio, G1011-10ML) for 5 to 10 min at room temperature, followed by PBS rinse. Hoechst was excited at 405 nm with sequential acquisition to avoid spectral bleed-through. For each condition, 3 to 5 nonoverlapping fields per coverslip were analyzed by blinded investigators; the mean value per coverslip was used for statistics. All dyes were prepared fresh on the day of use, and final solvent content in wells did not exceed 0.1%.

#### Assay allocation

Western blot samples were collected from 6-well plates; IF and colocalization (LC3B and TOM20, TOM20 and DEPP1) were performed on coverslips; TMRE assays for ΔΨm and viability assays were run in 96-well plates. All experiments were randomized and performed by investigators blinded to group allocation.

#### ARS and ALP

Calcium deposition and early osteogenic activity were assessed with commercial kits from Beyotime (Shanghai, China). For ARS staining of mineralized nodules, cells were cultured in osteogenic medium for 21 days, rinsed twice with PBS, fixed in 4 % paraformaldehyde for 15 min, and stained for 30 min at room temperature with the ARS working solution (pH 4.2; Cat. C0148S). Excess dye was removed by extensive washing with distilled water; plates were photographed under a light microscope, and bound dye was dissolved in 10% (w/v) cetyl-pyridinium chloride for 30 min, after which the optical density was read at 450 nm to obtain quantitative values of mineralization. ALP activity was examined on day 7 of induction with the BCIP/NBT chromogenic kit (Cat. C3206). After 2 PBS washes, cells were fixed for 10 min, incubated with freshly prepared BCIP/NBT working solution at 37 °C for 20 min in the dark until a blue-violet precipitate developed, washed with deionized water, and imaged.

### Molecular docking

Protein targets were identified in UniProt and corresponding experimental structures were downloaded from the Research Collaboratory for Structural Bioinformatics Protein Data Bank (RCSB PDB). The highest-quality entry covering the binding region was imported into Discovery Studio 2019 and prepared by removing nonessential crystallographic waters, adding hydrogens, assigning protonation states at pH 7.4, completing missing residues/side chains, assigning CHARMm charges, and performing a restrained energy minimization; the optimized receptor was exported as PDB. Small-molecule constituents of TFDF were retrieved from PubChem, converted to 3D, protonated for pH 7.4, minimized in Discovery Studio 2019, and saved as PDB. Receptor and ligands were converted to PDBQT in AutoDockTools 1.5.6 (Gasteiger charges; nonpolar hydrogens merged; ligand torsions defined). Docking was performed with AutoDock Vina 1.2.6 using a grid box centered on a co-crystallized ligand or a predicted pocket (margin ~5 to 8 Å per axis); typical parameters were exhaustiveness 8 to 16, num_modes 20, energy_range 4 kcal mol^−1^. Where available, redocking of the native ligand was used to validate the protocol (RMSD < 2.0 Å). Each ligand was docked in triplicate with different random seeds; pose selection considered Vina score, clustering, and chemically plausible interactions (hydrogen bonds, π–π/cation–π, hydrophobic contacts) with pocket residues. Complexes were visualized and annotated in PyMOL 3.1 and Discovery Studio 2019; grid coordinates and docking settings are reported in the Supplementary Methods.

### Molecular dynamics

Protein–ligand complexes were simulated with GROMACS 2025 for 100 ns. Protein parameters used AMBER99SB-ILDN; ligand topologies were built with GAFF2 and then merged with the protein to avoid atom-type conflicts. Periodic boundary conditions were applied in a cubic box with a 1.2-nm buffer from solute to box edge. The system was solvated with TIP3P water and neutralized with Na^+^/Cl^−^; salt concentration was set to 0.15 M. After NVT then NPT equilibration (total 2 ns, coupling time constant 0.1 ps), production runs were performed in the NPT ensemble at 310 K and 1 bar for 50,000,000 steps with a 2-fs timestep; thermostat/barostat settings matched equilibration. Trajectories were saved every 1,000 steps for analysis. Using GROMACS tools, we computed RMSD (global stability), RMSF (residue flexibility), radius of gyration, *R*_g_ (compactness), SASA (solvent exposure), and protein–ligand hydrogen-bond counts (interaction stability). Free-energy landscapes (2D/3D; Δ*G* = −*k*_B_*T* ln *P*) were constructed from principal components, and MM/PBSA was used to estimate mean binding free energy with per-residue energy decomposition.

### SPR analysis

SPR was used to characterize the direct binding between SIRT1 and naringenin using a CM5 sensor chip and standard amine-coupling chemistry. Immediately before use, the activation solution was prepared by mixing 400 mM EDC and 100 mM NHS. The carboxymethylated dextran surface of the CM5 chip was activated by injecting this EDC/NHS mixture at a flow rate of 10 μl/min for 420 s. Recombinant SIRT1 was diluted to 20 μg/ml in immobilization buffer and injected over the sample flow channel (Fc2) at 10 μl/min, typically yielding an immobilization level of approximately 12,600 response units (RU). The reference channel (Fc1) underwent the same activation and deactivation procedures without protein injection. Remaining active esters were then blocked by injecting 1 M ethanolamine hydrochloride at 10 μl/min for 420 s.

Multi-cycle measurements were performed with naringenin as the analyte. Naringenin was diluted in the analyte (running) buffer to 8 concentrations spanning 0.02 to 2 μM. Each concentration was injected sequentially over both flow channels (Fc1 and Fc2) at a flow rate of 20 μl/min with an association phase of 100 s, followed by a dissociation phase of 180 s in running buffer. After each injection cycle, the chip surface was regenerated with an appropriate regeneration solution (described in the Supplementary Methods), and a new cycle was initiated once a stable baseline was reestablished. Sensorgrams from the sample channel were reference-subtracted using the corresponding signal from the reference channel prior to further analysis.

### Study populations

The OP related genetic data come from the publicly available FinnGen database (Risteys FinnGen R6 - M13_OSTEOPOROSIS). The OP genome-wide association study (GWAS) data in the FinnGen biobank include 212,778 individuals of Finnish descent (3,203 cases and 209,575 controls). The MDD data are sourced from the UK Biobank and PGC (excluding 23andMe), encompassing 500,199 European individuals (170,756 cases and 329,443 controls). The GTEx V8 dataset contains extensive gene expression data from 49 different tissues, which were collected from 838 deceased donors (https://ftp.ebi.ac.uk/pub/databases/spot/eQTL/imported/GTEx_V8). The sample sizes vary across different tissues, ranging from 73 samples from the renal cortex to 706 samples from skeletal muscle.

### Gene-based association analysis

We utilized TWAS-fusion [54] to identify genes shared by the OP and MDD trait. In each method, the *P* value thresholds were adjusted through Bonferroni correction. TWAS identifies tissue-specific gene–trait associations by integrating GWAS with cis-SNV-based gene expression models [55]. We conducted TWAS using the FUSION software based on expression profiles from 43 postmortem tissues in GTEx. Cross-tissue transcriptome-wide association was further performed using S-MultiXcan, which integrates predicted gene expression across all GTEx V8 tissues to derive joint gene–trait association statistics for each phenotype. For each gene, S-MultiXcan *P* values were adjusted for multiple testing using the Bonferroni method, and genes surpassing the corrected threshold in both OP and MDD were considered shared candidates. To prioritize brain regions, we then conducted tissue enrichment and partitioned heritability analyses using LD score regression based on GTEx tissue-specific annotations, focusing on those tissues that showed significant enrichment after false discovery rate correction.

### Quantification and statistical analysis

The animal was the experimental unit for in vivo assays; independent culture (different passages/days) was the unit for cell assays. Data are presented as mean ± standard error of the mean (SEM) unless stated otherwise. Group sizes (*n*) and exact tests are reported in figure legends. Analyses were 2-tailed with *α* = 0.05. Normality was assessed by Shapiro–Wilk and homoscedasticity was assessed by Levene/Brown–Forsythe. For 2 groups, we used unpaired *t* tests (or Mann–Whitney *U* if nonnormal); for ≥3 groups, we used one-way analysis of variance (ANOVA) with Tukey (all-pairs) or Dunnett (vs. Model) post-hoc tests (or Kruskal–Wallis/Dunn nonparametric). Time-course data (e.g., body weight) used 2-way (repeated-measures) ANOVA with Greenhouse–Geisser correction if sphericity was violated. Outliers were handled a priori (surgical/behavioral exclusions defined in Materials and Methods/figure legends) and no data were removed post hoc. Statistics were run in GraphPad Prism 10 and R (v4.3).

## Data Availability

The OP GWAS summary statistics were obtained from the FinnGen biobank (release R6; endpoint M13_OSTEOPOROSIS). Public summary statistics can be downloaded from the FinnGen results portal (https://www.finngen.fi/en/access_results), and endpoint-level information can be explored through the Risteys browser (https://risteys.finngen.fi). MDD GWAS summary statistics were obtained from the Psychiatric Genomics Consortium (PGC) and the UK Biobank. PGC MDD summary statistics (excluding 23andMe) are available at https://pgc.unc.edu/for-researchers/download-results/, and UK Biobank genotype and phenotype data can be accessed upon approved application via the UK Biobank Data Showcase (https://biobank.ndph.ox.ac.uk/ukb/). Reference transcriptome and eQTL data for 49 tissues were obtained from the GTEx Project (v8 release) and are available through the GTEx Portal (https://gtexportal.org/home/). TWAS analyses were performed with the FUSION software, which, together with reference weight panels, is freely available from the Gusev Lab website (http://gusevlab.org/projects/fusion/), and cross-tissue S-MultiXcan analyses were carried out using the MetaXcan framework (https://github.com/hakyimlab/MetaXcan).

## References

[1] Newman M, Donahue HJ, Neigh GN. Connecting the dots: Sex, depression, and musculoskeletal health. J Clin Invest. 2024;134(18): Article e180072. 39286983 10.1172/JCI180072 PMC11405046

[2] Qiu L, Yang Q, Sun N, Li D, Zhao Y, Li X, Gong Y, Lv C, Yin X. Association between depression and the risk for fracture: A meta-analysis and systematic review. BMC Psychiatry. 2018;18(1):336. 30333001 10.1186/s12888-018-1909-2 PMC6192066

[3] Cheng CH, Chen LR, Chen KH. Osteoporosis due to hormone imbalance: An overview of the effects of estrogen deficiency and glucocorticoid overuse on bone turnover. Int J Mol Sci. 2022;23(3):1376. 35163300 10.3390/ijms23031376 PMC8836058

[4] Khosla S, Melton LJ III, Riggs BL. The unitary model for estrogen deficiency and the pathogenesis of osteoporosis: Is a revision needed? J Bone Miner Res. 2011;26(3):441–451. 20928874 10.1002/jbmr.262 PMC3179298

[5] Wang J, Zhang Y, Cao J, Wang Y, Anwar N, Zhang Z, Zhang D, Ma Y, Xiao Y, Xiao L, et al. The role of autophagy in bone metabolism and clinical significance. Autophagy. 2023;19(9):2409–2427. 36858962 10.1080/15548627.2023.2186112 PMC10392742

[6] Liu J, Gao Z, Liu X. Mitochondrial dysfunction and therapeutic perspectives in osteoporosis. Front Endocrinol. 2024;15:1325317. 10.3389/fendo.2024.1325317 PMC10870151 38370357

[7] Lips P, Cooper C, Agnusdei D, Caulin F, Egger P, Johnell O, Kanis JA, Kellingray S, Leplege A, Liberman UA, et al. Quality of life in patients with vertebral fractures: Validation of the quality of life questionnaire of the European Foundation for osteoporosis (QUALEFFO). Working Party for Quality of life of the European Foundation for osteoporosis. Osteoporos Int. 1999;10(2):150–160. 10501796 10.1007/s001980050210

[8] Chen K, Wang T, Tong X, Song Y, Hong J, Sun Y, Zhuang Y, Shen H, Yao XI. Osteoporosis is associated with depression among older adults: A nationwide population-based study in the USA from 2005 to 2020. Public Health. 2024;226:27–31. 37988825 10.1016/j.puhe.2023.10.022

[9] Berk M, Köhler-Forsberg O, Turner M, Penninx BWJH, Wrobel A, Firth J, Loughman A, Reavley NJ, McGrath J, Momen NC, et al. Comorbidity between major depressive disorder and physical diseases: A comprehensive review of epidemiology, mechanisms and management. World Psychiatry. 2023;22(3):366–387. 37713568 10.1002/wps.21110 PMC10503929

[10] Tops L, Beerten SG, Vandenbulcke M, Vermandere M, Deschodt M. Integrated care models for older adults with depression and physical comorbidity: A scoping review. Int J Integr Care. 2024;24(1):1. 10.5334/ijic.7576 PMC10786096 38222854

[11] Cummings SR, San Martin J, McClung M, Siris ES, Eastell R, Reid IR, Delmas P, Zoog HB, Austin M, Wang A, et al. Denosumab for prevention of fractures in postmenopausal women with osteoporosis. N Engl J Med. 2009;361(8):756–765. 19671655 10.1056/NEJMoa0809493

[12] Cauley JA, Robbins J, Chen Z, Cummings SR, Jackson RD, LaCroix AZ, LeBoff M, Lewis CE, McGowan J, Neuner J, et al. Effects of estrogen plus progestin on risk of fracture and bone mineral density: The Women’s Health Initiative Randomized Trial. JAMA. 2003;290(13):1729–1738. 14519707 10.1001/jama.290.13.1729

[13] Rossouw JE, Anderson GL, Prentice RL, LaCroix A, Kooperberg C, Stefanick ML, Jackson RD, Beresford SA, Howard BV, Johnson KC, et al. Risks and benefits of estrogen plus progestin in healthy postmenopausal women: Principal results from the Women’s Health Initiative randomized controlled trial. JAMA. 2002;288(3):321–333. 12117397 10.1001/jama.288.3.321

[14] Ettinger B, Black DM, Mitlak BH, Knickerbocker RK, Nickelsen T, Genant HK, Christiansen C, Delmas PD, Zanchetta JR, Stakkestad J, et al. Reduction of vertebral fracture risk in postmenopausal women with osteoporosis treated with raloxifene: Results from a 3-year randomized clinical trial. Multiple outcomes of Raloxifene evaluation (MORE) investigators. JAMA. 1999;282(7):637–645. 10517716 10.1001/jama.282.7.637

[15] Zhang Y, Jiang J, Shen H, Chai Y, Wei X, Xie Y. Total flavonoids from Rhizoma Drynariae (Gusuibu) for treating osteoporotic fractures: Implication in clinical practice. Drug Des Devel Ther. 2017;11:1881–1890. 10.2147/DDDT.S139804 PMC5491704 28694688

[16] Shen Z, Dong W, Chen Z, Chen G, Zhang Y, Li Z, Lin H, Chen H, Huang M, Guo Y, et al. Total flavonoids of Rhizoma Drynariae enhances CD31(hi)Emcn(hi) vessel formation and subsequent bone regeneration in rat models of distraction osteogenesis by activating PDGF-BB/VEGF/RUNX2/OSX signaling axis. Int J Mol Med. 2022;50(3):112. 35795995 10.3892/ijmm.2022.5167 PMC9330352

[17] Li F, Sun X, Ma J, Ma X, Zhao B, Zhang Y, Tian P, Li Y, Han Z. Naringin prevents ovariectomy-induced osteoporosis and promotes osteoclasts apoptosis through the mitochondria-mediated apoptosis pathway. Biochem Biophys Res Commun. 2014;452(3):629–635. 25181344 10.1016/j.bbrc.2014.08.117

[18] Gan J, Deng X, Le Y, Lai J, Liao X. The development of Naringin for use against bone and cartilage disorders. Molecules. 2023;28(9):3716. 37175126 10.3390/molecules28093716 PMC10180405

[19] Gao C, Wu M, Du Q, Deng J, Shen J. Naringin mediates adult hippocampal neurogenesis for antidepression via activating CREB signaling. Front Cell Dev Biol. 2022;10: Article 731831. 35478969 10.3389/fcell.2022.731831 PMC9037031

[20] Song S, Gao Z, Lei X, Niu Y, Zhang Y, Li C, Lu Y, Wang Z, Shang P. Total flavonoids of Drynariae Rhizoma prevent bone loss induced by hindlimb unloading in rats. Molecules. 2017;22(7):1033. 28640230 10.3390/molecules22071033 PMC6152118

[21] Zhang L, Cao LL, Yang DD, Ding JH, Guo XD, Xue TF, Zhao XJ, Sun XL. Establishment and evaluation of a novel mouse model of peri/postmenopausal depression. Heliyon. 2019;5(2): Article e01195. 30839939 10.1016/j.heliyon.2019.e01195 PMC6365542

[22] Hariharan N, Maejima Y, Nakae J, Paik J, Depinho RA, Sadoshima J. Deacetylation of FoxO by Sirt1 plays an essential role in mediating starvation-induced autophagy in cardiac myocytes. Circ Res. 2010;107(12):1470–1482. 20947830 10.1161/CIRCRESAHA.110.227371 PMC3011986

[23] Liu BH, Xu CZ, Liu Y, Lu ZL, Fu TL, Li GR, Deng Y, Luo GQ, Ding S, Li N, et al. Mitochondrial quality control in human health and disease. Mil Med Res. 2024;11(1):32. 38812059 10.1186/s40779-024-00536-5 PMC11134732

[24] Michán S, Li Y, Chou MM, Parrella E, Ge H, Long JM, Allard JS, Lewis K, Miller M, Xu W, et al. SIRT1 is essential for normal cognitive function and synaptic plasticity. J Neurosci. 2010;30(29):9695–9707. 20660252 10.1523/JNEUROSCI.0027-10.2010 PMC2921958

[25] Artsi H, Cohen-Kfir E, Gurt I, Shahar R, Bajayo A, Kalish N, Bellido TM, Gabet Y, Dresner-Pollak R. The Sirtuin1 activator SRT3025 down-regulates sclerostin and rescues ovariectomy-induced bone loss and biomechanical deterioration in female mice. Endocrinology. 2014;155(9):3508–3515. 24949665 10.1210/en.2014-1334 PMC11651357

[26] Wang C, Chen R, Zhu X, Zhang X, Lian N. METTL14 alleviates the development of osteoporosis in ovariectomized mice by upregulating m(6)a level of SIRT1 mRNA. Bone. 2023;168: Article 116652. 36584783 10.1016/j.bone.2022.116652

[27] Zhu C, Shen S, Zhang S, Huang M, Zhang L, Chen X. Autophagy in bone remodeling: A regulator of oxidative stress. Front Endocrinol. 2022;13: Article 898634. 10.3389/fendo.2022.898634 PMC9279723 35846332

[28] Gassen NC, Rein T. Is there a role of autophagy in depression and antidepressant action? Front Psych. 2019;10:337. 10.3389/fpsyt.2019.00337 PMC6529564 31156481

[29] Li L, Gu L, Hua Y, Xu H, Ling Y, Tong L, Chen M, Ma R. Kumatakenin alleviates depressive-like behaviors by suppressing excessive autophagy in hippocampus via ATG5. Eur J Pharmacol. 2025;999: Article 177688. 40294778 10.1016/j.ejphar.2025.177688

[30] Chen L, Lu L, Fan C, Zhu X, Pan L, Tang S, Wang Y, Peng Y, You L. Autophagy-induced osteoblast-derived exosomes maintain bone formation and prevent osteoporosis by remodeling gut microbiota-metabolism. Biom J. 2025; Article 100870. 10.1016/j.bj.2025.100870 PMC12886068 40339904

[31] Qiu X, Pan T, Kuang T, Shen Y, Zheng Y, Geng H, Ni B, Xia X, Zhu C, Zhang Z, et al. DEPP1: A prognostic biomarker linked to stroma-rich and immunosuppressive microenvironment, promoting oxaliplatin resistance in gastric cancer. Oncol Rep. 2025;54(1):82. 40376989 10.3892/or.2025.8915 PMC12117316

[32] Patel S, Naik L, das M, Nayak DK, Dandsena PK, Mishra A, Kumar A, Dirisala VR, Mishra A, das S, et al. Furamidine-induced autophagy exerts an anti-mycobacterial effect in a SIRT1-pAMPK-FOXO3a-dependent manner by elevation of intracellular Ca^2+^ level expression. Microbiol Res. 2025;290: Article 127976. 39591744 10.1016/j.micres.2024.127976

[33] Yan D, Yang Y, Lang J, Wang X, Huang Y, Meng J, Wu J, Zeng X, Li H, Ma H, et al. SIRT1/FOXO3-mediated autophagy signaling involved in manganese-induced neuroinflammation in microglia. Ecotoxicol Environ Saf. 2023;256: Article 114872. 37027942 10.1016/j.ecoenv.2023.114872

[34] Xu C, Liao M, Zhang S, Chen Y, Shulai X, Wang G, Aa J. The comorbidity of depression and diabetes is involved in the Decidual protein induced by progesterone 1 (Depp1) dysfunction in the medial prefrontal cortex. Meta. 2025;15(1):34. 10.3390/metabo15010034 PMC11767987 39852377

[35] Wong KC, Pang WY, Wang XL, Mok SK, Lai WP, Chow HK, Leung PC, Yao XS, Wong MS. Drynaria fortunei-derived total flavonoid fraction and isolated compounds exert oestrogen-like protective effects in bone. Br J Nutr. 2013;110(3):475–485. 23302510 10.1017/S0007114512005405

[36] Ahn JS, Lee CH, Liu XQ, Hwang KW, Oh MH, Park SY, Whang WK. Neuroprotective effects of phenolic constituents from Drynariae Rhizoma. Pharmaceuticals. 2024;17(8):1061. 39204166 10.3390/ph17081061 PMC11358882

[37] Li X, Wichai N, Wang J, Liu X, Yan H, Wang Y, Luo M, Zhou S, Wang K, Li L, et al. Regulation of innate and adaptive immunity using herbal medicine: Benefits for the COVID-19 vaccination. Acupunct Herb Med. 2022;2(3):196–206. 37808346 10.1097/HM9.0000000000000046 PMC9746255

[38] Chen R, Gao S, Guan H, Zhang X, Gao Y, Su Y, Song Y, Jiang Y, Li N. Naringin protects human nucleus pulposus cells against TNF-α-induced inflammation, oxidative stress, and loss of cellular homeostasis by enhancing autophagic flux via AMPK/SIRT1 activation. Oxidative Med Cell Longev. 2022;2022:7655142. 10.1155/2022/7655142 PMC8898769 35265264

[39] Kang L, Zhang H, Jia C, Zhang R, Shen C. Targeting oxidative stress and inflammation in intervertebral disc degeneration: Therapeutic perspectives of phytochemicals. Front Pharmacol. 2022;13: Article 956355. 35903342 10.3389/fphar.2022.956355 PMC9315394

[40] Brunet A, Sweeney LB, Sturgill JF, Chua KF, Greer PL, Lin Y, Tran H, Ross SE, Mostoslavsky R, Cohen HY, et al. Stress-dependent regulation of FOXO transcription factors by the SIRT1 deacetylase. Science. 2004;303(5666):2011–2015. 14976264 10.1126/science.1094637

[41] Salama AAA, Yassen NN, Mansour HM. Naringin protects mice from D-galactose-induced lung aging and mitochondrial dysfunction: Implication of SIRT1 pathways. Life Sci. 2023;324: Article 121471. 36746356 10.1016/j.lfs.2023.121471

[42] Wu Y, Liu H, Wang Q, Zhang T, Chen R, Yuan Q, Tong X, Yang W, Xiao Y, Yan F. Neoeriocitrin targeting Beclin1 deubiquitination and autophagy in osteogenic differentiation of human dental pulp stem cells. Adv Sci. 2025;12(43): Article e04378. 10.1002/advs.202504378 PMC12631872 40831238

[43] Wang G, Yang H, Zuo W, Mei X. Antidepressant-like effect of acute dose of Naringin involves suppression of NR1 and activation of protein kinase A/cyclic adenosine monophosphate response element-binding protein/brain-derived neurotrophic factor signaling in hippocampus. Behav Pharmacol. 2023;34(2–3):101–111. 36503881 10.1097/FBP.0000000000000713

[44] Wang X, Chen L, Peng W. Protective effects of resveratrol on osteoporosis via activation of the SIRT1-NF-κB signaling pathway in rats. Exp Ther Med. 2017;14(5):5032–5038. 29201210 10.3892/etm.2017.5147 PMC5704326

[45] Zainabadi K. Drugs targeting SIRT1, a new generation of therapeutics for osteoporosis and other bone related disorders? Pharmacol Res. 2019;143:97–105. 30862606 10.1016/j.phrs.2019.03.007

[46] Kodali M, Attaluri S, Madhu LN, Shuai B, Upadhya R, Gonzalez JJ, Rao X, Shetty AK. Metformin treatment in late middle age improves cognitive function with alleviation of microglial activation and enhancement of autophagy in the hippocampus. Aging Cell. 2021;20(2): Article e13277. 33443781 10.1111/acel.13277 PMC7884047

[47] Sun B, Yin YZ, Xiao J. An in vivo estrogen deficiency mouse model for screening exogenous estrogen treatments of cardiovascular dysfunction after menopause. J Vis Exp. 2019; 10.3791/59536. 10.3791/59536 31475965

[48] Leng L, Zhuang K, Liu Z, Huang C, Gao Y, Chen G, Lin H, Hu Y, Wu D, Shi M, et al. Menin deficiency leads to depressive-like behaviors in mice by modulating astrocyte-mediated neuroinflammation. Neuron. 2018;100(3):551–563.e7. 30220511 10.1016/j.neuron.2018.08.031

[49] Zhang K, Wang Z, Pan X, Yang J, Wu C. Antidepressant-like effects of Xiaochaihutang in perimenopausal mice. J Ethnopharmacol. 2020;248: Article 112318. 31629860 10.1016/j.jep.2019.112318

[50] Sun N, Qin YJ, Xu C, Xia T, du ZW, Zheng LP, Li AA, Meng F, Zhang Y, Zhang J, et al. Design of fast-onset antidepressant by dissociating SERT from nNOS in the DRN. Science. 2022;378(6618):390–398. 36302033 10.1126/science.abo3566

[51] Liu MY, Yin CY, Zhu LJ, Zhu XH, Xu C, Luo CX, Chen H, Zhu DY, Zhou QG. Sucrose preference test for measurement of stress-induced anhedonia in mice. Nat Protoc. 2018;13(7):1686–1698. 29988104 10.1038/s41596-018-0011-z

[52] Dai SH, Chen T, Wang YH, Zhu J, Luo P, Rao W, Yang YF, Fei Z, Jiang XF. Sirt3 attenuates hydrogen peroxide-induced oxidative stress through the preservation of mitochondrial function in HT22 cells. Int J Mol Med. 2014;34(4):1159–1168. 25090966 10.3892/ijmm.2014.1876

[53] Fatokun AA, Stone TW, Smith RA. Hydrogen peroxide-induced oxidative stress in MC3T3-E1 cells: The effects of glutamate and protection by purines. Bone. 2006;39(3):542–551. 16616712 10.1016/j.bone.2006.02.062

[54] Gusev A, Ko A, Shi H, Bhatia G, Chung W, Penninx BW, Jansen R, de Geus EJ, Boomsma DI, Wright FA, et al. Integrative approaches for large-scale transcriptome-wide association studies. Nat Genet. 2016;48(3):245–252. 26854917 10.1038/ng.3506 PMC4767558

[55] Gamazon ER, Wheeler HE, Shah KP, Mozaffari SV, Aquino-Michaels K, Carroll RJ, Eyler AE, Denny JC, GTEx Consortium, Nicolae DL, et al. A gene-based association method for mapping traits using reference transcriptome data. Nat Genet. 2015;47(9):1091–1098. 26258848 10.1038/ng.3367 PMC4552594

