# Supplementary material for: Targeting a Shared Mitophagy Regulator: The SIRT1–FOXO3–DEPP1 Axis Underpins the Dual Bone and Brain Benefits of Total Flavonoids from Drynaria fortunei
Source: Research (Wash D C). 2026 Feb 24;9:1125. doi: 10.34133/research.1125 (PMC12929817; doi:10.34133/research.1125)
Supplement: Supplementary 1 — Figs. S1 to S8 Tables S1 to S3 [file research.1125.f1.docx]

**Table S1 siRNA oligonucleotides targeting mouse Sirt1 and Depp1, and a non‑targeting control.**

| **Target gene** | **Assay** | **Sense (5’→3’)** | **Antisense (5’→3’)** | **Type** | **Final conc.** |
| --- | --- | --- | --- | --- | --- |
| **Sirt1** | siRNA‑#1 | GACAAAGCUGAAGCCUUUA **dTdT** | UAAAGGCUUCAGCUUUGUC **dTdT** | siRNA | 50 nM |
| **Sirt1** | siRNA‑#2 | GGAUAGGUUCCUGAUAAUA **dTdT** | UAUUAUCAGGAACCUAUCC **dTdT** | siRNA | 50 nM |
| **Depp1** | siRNA‑#1 | GGACCUUGAUGAAGAAAU A **dTdT** | UAUUUCUUCAUCAAGGUCC **dTdT** | siRNA | 50 nM |
| **Depp1** | siRNA‑#2 | CCACCUAUGUCAUCUUAAA **dTdT** | UUU AAGAUGACA UAGGUGG **dTdT** | siRNA | 50 nM |
| **Negative control** | siRNA‑NC | UUCUCCGAACGUGUCACGU **dTdT** | ACGUGACACGUUCGGAGAA **dTdT** | siRNA | 50 nM |

This table lists the duplex sequences used for transient knockdown in HT‑22 and MC3T3‑E1 cells. Two non‑overlapping siRNAs were designed for each gene; all duplexes carry 3′ dTdT overhangs and were applied at a final concentration of 50 nM unless noted. Sense and antisense strands are written 5′→3′. A scrambled siRNA (siRNA‑NC) served as the negative control. Knockdown efficiency was verified by RT‑qPCR and Western blot 36–48 h post‑transfection.

**Table S2 Transcriptome‑wide association study (TWAS) signals for OP and MDD across GTEx brain panels.**

|  | **PANEL.x** | **MODELCV.R2.x** | **MODELCV.PV.x** | **TWAS.P.fdr.x** | **MODELCV.R2.y** | **MODELCV.PV.y** | **TWAS.P.fdr.y** |
| --- | --- | --- | --- | --- | --- | --- | --- |
| ENSG00000075413 | Anterior_cingulate_cortex_BA24 | 0.0071 | 0.15 | 3.65E-05 | 0.062 | 0.00019 | 0.01010963 |
| ENSG00000075413 | Anterior_cingulate_cortex_BA24 | 0.0071 | 0.15 | 3.65E-05 | 0.088 | 3.80E-05 | 0.01199682 |
| ENSG00000126214 | Caudate_basal_ganglia | 0.011 | 0.081 | 4.84E-05 | 0.011 | 0.081 | 0.0217874 |
| ENSG00000126214 | Cerebellar_Hemisphere | 0.15 | 7.00E-08 | 1.12E-05 | 0.011 | 0.081 | 0.0217874 |
| ENSG00000166166 | Cortex | 0.086 | 1.20E-05 | 1.55E-06 | 0.061 | 0.00029 | 0.01718193 |
| ENSG00000166166 | Cortex | 0.086 | 1.20E-05 | 1.55E-06 | 0.086 | 1.20E-05 | 0.00023713 |
| ENSG00000166166 | Cortex | 0.086 | 1.20E-05 | 1.55E-06 | 0.1 | 1.10E-05 | 0.01199682 |
| ENSG00000166166 | Cortex | 0.086 | 1.20E-05 | 1.55E-06 | 0.045 | 0.0014 | 0.00974345 |
| ENSG00000166166 | Frontal_Cortex_BA9 | 0.1 | 1.10E-05 | 1.45E-07 | 0.061 | 0.00029 | 0.01718193 |
| ENSG00000166166 | Frontal_Cortex_BA9 | 0.1 | 1.10E-05 | 1.45E-07 | 0.086 | 1.20E-05 | 0.00023713 |
| ENSG00000166166 | Frontal_Cortex_BA9 | 0.1 | 1.10E-05 | 1.45E-07 | 0.1 | 1.10E-05 | 0.01199682 |
| ENSG00000166166 | Frontal_Cortex_BA9 | 0.1 | 1.10E-05 | 1.45E-07 | 0.045 | 0.0014 | 0.00974345 |
| ENSG00000166166 | Hippocampus | 0.037 | 0.008 | 0.00216104 | 0.061 | 0.00029 | 0.01718193 |
| ENSG00000166166 | Hippocampus | 0.037 | 0.008 | 0.00216104 | 0.086 | 1.20E-05 | 0.00023713 |
| ENSG00000166166 | Hippocampus | 0.037 | 0.008 | 0.00216104 | 0.1 | 1.10E-05 | 0.01199682 |
| ENSG00000166166 | Hippocampus | 0.037 | 0.008 | 0.00216104 | 0.045 | 0.0014 | 0.00974345 |
| ENSG00000166170 | Anterior_cingulate_cortex_BA24 | 0.13 | 4.30E-06 | 0.0001139 | 0.12 | 9.60E-07 | 0.02277317 |
| ENSG00000166170 | Cerebellar_Hemisphere | 0.12 | 9.60E-07 | 9.82E-07 | 0.12 | 9.60E-07 | 0.02277317 |
| ENSG00000166170 | Hypothalamus | 0.12 | 3.00E-06 | 1.48E-06 | 0.12 | 9.60E-07 | 0.02277317 |
| ENSG00000172260 | Caudate_basal_ganglia | 0.093 | 8.80E-06 | 5.07E-14 | 0.093 | 8.80E-06 | 0.01718193 |
| ENSG00000172260 | Caudate_basal_ganglia | 0.093 | 8.80E-06 | 5.07E-14 | 0.18 | 3.00E-09 | 0.03990325 |
| ENSG00000172260 | Nucleus_accumbens_basal_ganglia | 0.09 | 8.60E-06 | 1.12E-11 | 0.093 | 8.80E-06 | 0.01718193 |
| ENSG00000172260 | Nucleus_accumbens_basal_ganglia | 0.09 | 8.60E-06 | 1.12E-11 | 0.18 | 3.00E-09 | 0.03990325 |
| ENSG00000172260 | Putamen_basal_ganglia | 0.18 | 3.00E-09 | 1.29E-10 | 0.093 | 8.80E-06 | 0.01718193 |
| ENSG00000172260 | Putamen_basal_ganglia | 0.18 | 3.00E-09 | 1.29E-10 | 0.18 | 3.00E-09 | 0.03990325 |
| ENSG00000227207 | Cerebellar_Hemisphere | 0.49 | 4.90E-27 | 8.62E-14 | 0.49 | 4.90E-27 | 0.02277317 |
| ENSG00000258851 | Cerebellar_Hemisphere | 0.084 | 6.20E-05 | 8.36E-08 | 0.084 | 6.20E-05 | 0.03831 |
| ENSG00000261543 | Cortex | 0.21 | 3.90E-12 | 0.03179715 | 0.21 | 3.90E-12 | 0.036301 |
| ENSG00000261543 | Frontal_Cortex_BA9 | 0.25 | 1.60E-12 | 0.0301125 | 0.21 | 3.90E-12 | 0.036301 |
| ENSG00000278376 | Anterior_cingulate_cortex_BA24 | 0.31 | 2.20E-13 | 0.00750682 | 0.5 | 3.70E-28 | 0.03831 |
| ENSG00000278376 | Anterior_cingulate_cortex_BA24 | 0.31 | 2.20E-13 | 0.00750682 | 0.29 | 2.50E-14 | 0.034808 |
| ENSG00000278376 | Caudate_basal_ganglia | 0.35 | 1.20E-19 | 0.03278468 | 0.5 | 3.70E-28 | 0.03831 |
| ENSG00000278376 | Caudate_basal_ganglia | 0.35 | 1.20E-19 | 0.03278468 | 0.29 | 2.50E-14 | 0.034808 |
| ENSG00000278376 | Cerebellar_Hemisphere | 0.5 | 3.70E-28 | 0.01445026 | 0.5 | 3.70E-28 | 0.03831 |
| ENSG00000278376 | Cerebellar_Hemisphere | 0.5 | 3.70E-28 | 0.01445026 | 0.29 | 2.50E-14 | 0.034808 |
| ENSG00000278376 | Hippocampus | 0.34 | 1.40E-16 | 0.01695755 | 0.5 | 3.70E-28 | 0.03831 |
| ENSG00000278376 | Hippocampus | 0.34 | 1.40E-16 | 0.01695755 | 0.29 | 2.50E-14 | 0.034808 |
| ENSG00000278376 | Nucleus_accumbens_basal_ganglia | 0.4 | 1.90E-24 | 0.04213798 | 0.5 | 3.70E-28 | 0.03831 |
| ENSG00000278376 | Nucleus_accumbens_basal_ganglia | 0.4 | 1.90E-24 | 0.04213798 | 0.29 | 2.50E-14 | 0.034808 |
| ENSG00000278376 | Putamen_basal_ganglia | 0.29 | 2.50E-14 | 0.00689694 | 0.5 | 3.70E-28 | 0.03831 |
| ENSG00000278376 | Putamen_basal_ganglia | 0.29 | 2.50E-14 | 0.00689694 | 0.29 | 2.50E-14 | 0.034808 |
| ENSG00000278376 | Substantia_nigra | 0.3 | 1.60E-10 | 0.038537 | 0.5 | 3.70E-28 | 0.03831 |
| ENSG00000278376 | Substantia_nigra | 0.3 | 1.60E-10 | 0.038537 | 0.29 | 2.50E-14 | 0.034808 |

The table lists TWAS results for genes (Ensembl IDs) whose predicted expression (built from GTEx reference models) associates with osteoporosis (OP; columns ending in “.x”) and major depressive disorder (MDD; columns ending in “.y”) in specific brain tissues (“PANEL”). MODELCV.R2 and MODELCV.PV report cross‑validation performance of the expression model for each tissue; TWAS.P.fdr is the FDR‑adjusted association P value for the trait. Several genes show convergent associations across multiple regions, with replicating hits in cortex and frontal cortex (e.g., repeated entries for ENSG00000166166) and consistent signals in the hippocampus (e.g., hippocampal FDR for OP as low as ~2.2×10⁻³ and for MDD as low as ~2.4×10⁻⁴ for the same gene), supporting a shared hippocampal contribution to OP–MDD comorbidity. Additional loci appear in striatal nuclei (caudate, putamen, nucleus accumbens), where OP associations are often extremely strong (OP FDR down to ~10⁻¹⁴) with parallel but weaker MDD signals. Overall, model CV performance was acceptable (R² typically 0.03–0.50), and the concentration of low FDR values in hippocampus and cortex motivated hippocampus‑focused experimental validation in the main text.


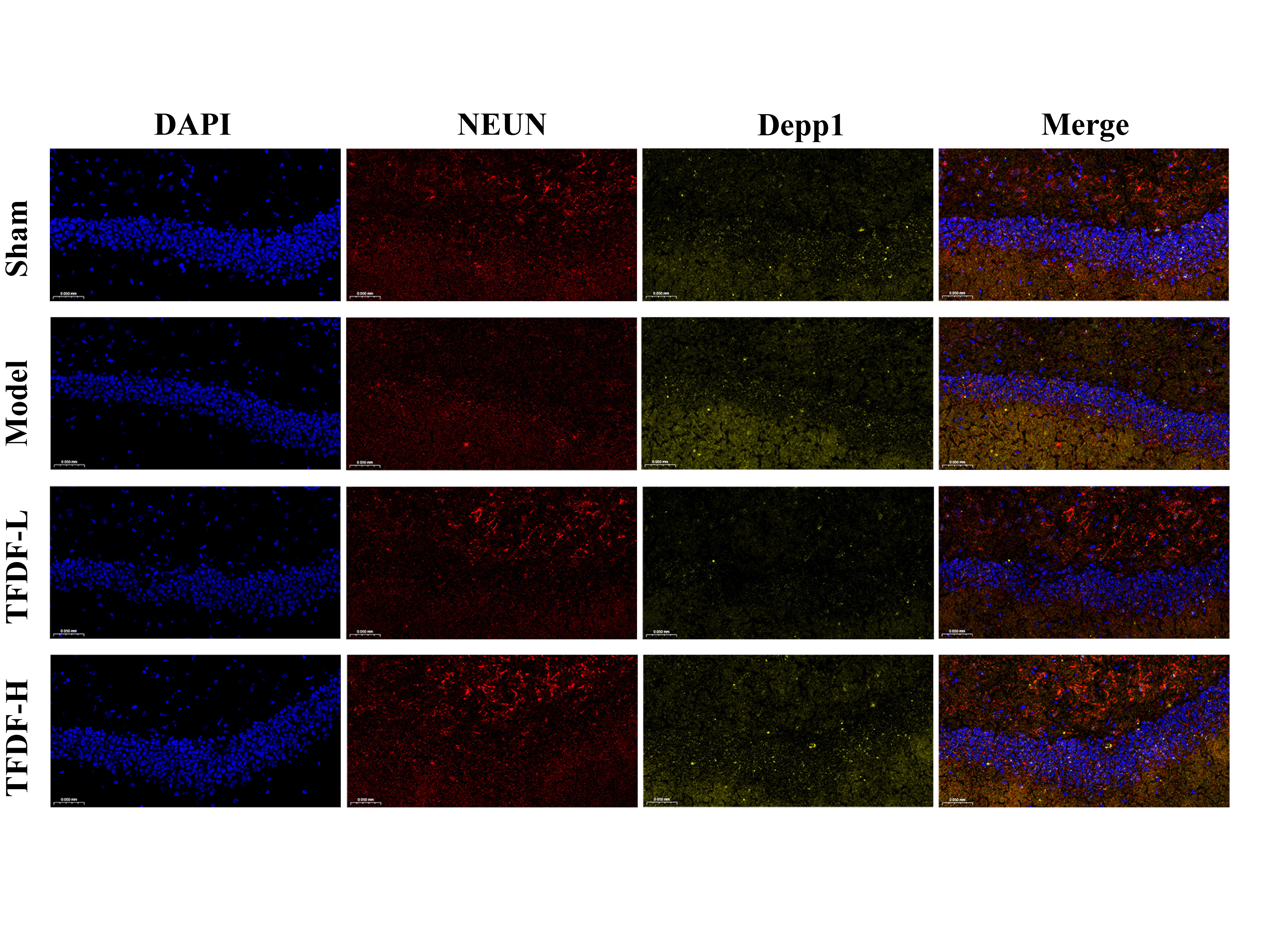


**Figure S1 Dentate gyrus (DG) immunofluorescence shows TFDF lowers neuronal DEPP1 in a dose‑dependent manner.**

Representative confocal images from the hippocampal DG across groups (Sham, OVX–CUMS Model, TFDF‑L, TFDF‑H). Nuclei are labeled with DAPI (blue), neurons with NeuN (red), and DEPP1 (yellow); merged panels are shown at right. Compared with Sham, OVX–CUMS mice display stronger, punctate DEPP1 staining within NeuN‑positive layers and a sparser granule cell band. TFDF reduces neuronal DEPP1 signal intensity in a dose‑responsive fashion and visibly improves granule‑cell layer organization. Images were acquired under identical settings; scale bars as indicated.


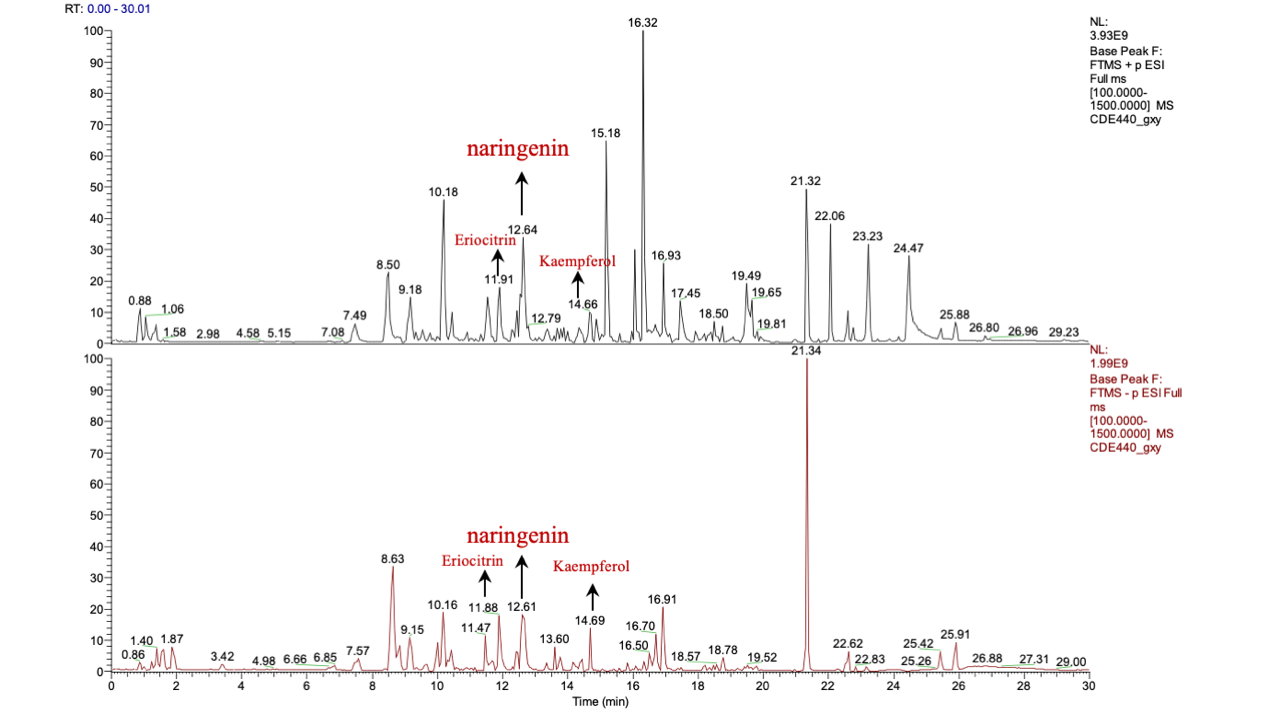


**Fig. S2. UPLC–Q-Orbitrap HRMS chromatograms of TFDF powder.**

Base-peak chromatograms of TFDF in (upper) positive-ion and (lower) negative-ion ESI modes over 0–30 min. Peak labels indicate retention times (min); assignments of major flavonoids (naringin, naringenin chalcone, naringenin, eriodictyol, astragalin, kaempferol-7-O-glucoside, etc.), phenolic acids, procyanidin oligomers and azelaic acid. Naringenin elutes at approximately 12.56 min.
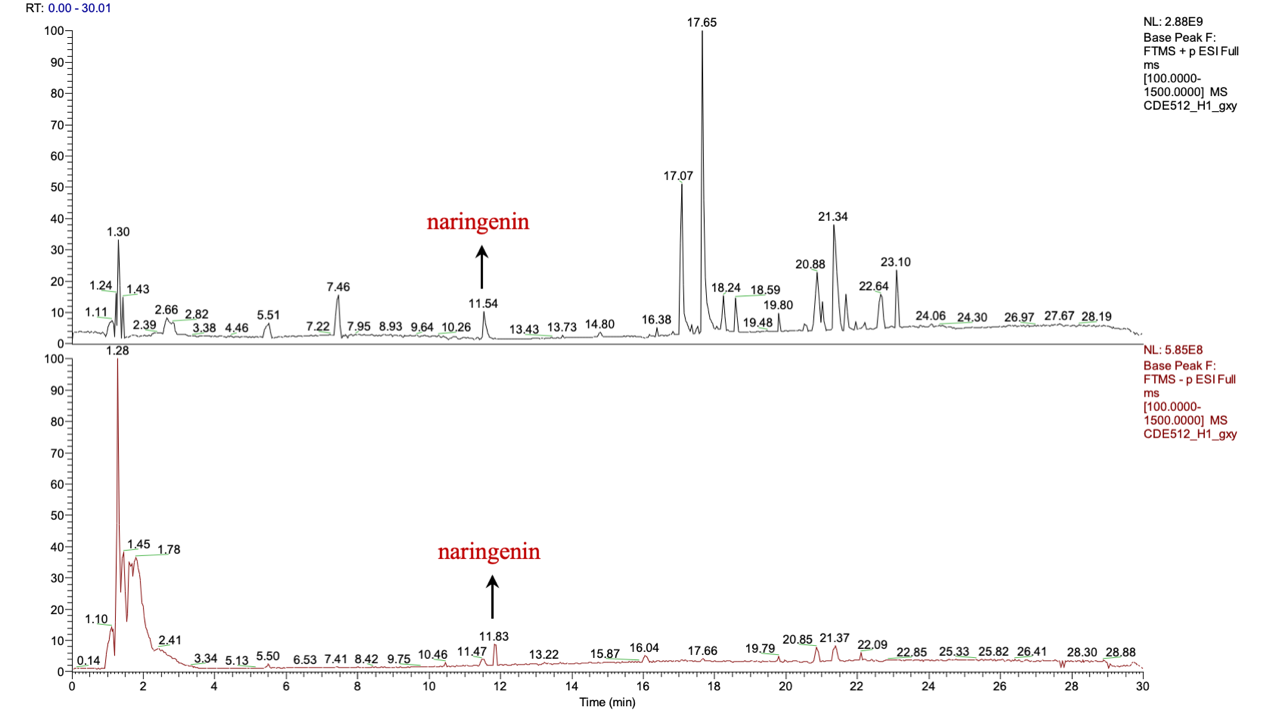


**Fig S3 UPLC–Q-Orbitrap HRMS chromatograms of serum from TFDF-treated OVX–CUMS mice.**

Base-peak chromatograms acquired in (upper) positive-ion and (lower) negative-ion ESI modes. The traces show a complex endogenous metabolite background (amino acids, carnitines, bile acids, lysophosphatidylcholines and eicosanoids) overlaid with a subset of TFDF-derived small molecules. Peak labels indicate retention times (min), and putative assignments, including azelaic acid and several long-chain fatty-acid–related species.

**Table S3 Comparative docking of TFDF constituents with DEPP1 and SIRT1**

| **Ligand** | **Binding Affinity** | **rmsd/ub** | **rmsd/lb** |
| --- | --- | --- | --- |
| **DEPP1-Naringenin** | -5.4 | 5.463 | 2.791 |
| **DEPP1-Naringin** | -5.9 | 38.387 | 33.272 |
| **DEPP1-Neoeriocitrin** | -5.8 | 28.705 | 24.712 |
| **DEPP1-Etoposide** | -6.2 | 1.923 | 0.442 |
| **SIRT1-Naringenin** | -6.8 | 6.633 | 2.559 |
| **SIRT1-Naringin** | -8 | 3.26 | 2.131 |
| **SIRT1-Neoeriocitrin** | -8.5 | 3.343 | 2.119 |
| **SIRT1-Resveratrol** | -6.9 | 12.997 | 11.601 |

AutoDock Vina docking showed that the three major TFDF constituents (naringenin, naringin, neoeriocitrin) had weak affinity for DEPP1 (−5.4 to −5.9 kcal mol⁻¹) and non‑convergent poses (large rmsd l.b./u.b., typically >20 Å), consistent with poor compatibility of these ligands with the putative DEPP1 pockets. A reference ligand (etoposide) returned a slightly better score (−6.2 kcal mol⁻¹) and convergent docking (rmsd l.b./u.b. ≈ 0.44/1.92 Å), supporting the validity of the docking protocol.

In contrast, SIRT1 displayed stronger and more coherent binding to TFDF flavonoids: neoeriocitrin and naringin scored −8.5 and −8.0 kcal mol⁻¹, respectively, and naringenin −6.8 kcal mol⁻¹, with moderate rmsd l.b./u.b. values (≈2–6 Å) indicating defined binding modes. The positive control resveratrol scored −6.9 kcal mol⁻¹ but showed higher pose dispersion (rmsd ≈ 11–13 Å).

Together, these results support SIRT1 as the preferred direct binding target of TFDF constituents relative to DEPP1. (Notes: Binding Affinity is the Vina score in kcal mol⁻¹; more negative values indicate stronger predicted binding. rmsd l.b./u.b. are lower/upper‑bound RMSDs to the top‑ranked pose; small values denote pose convergence.)


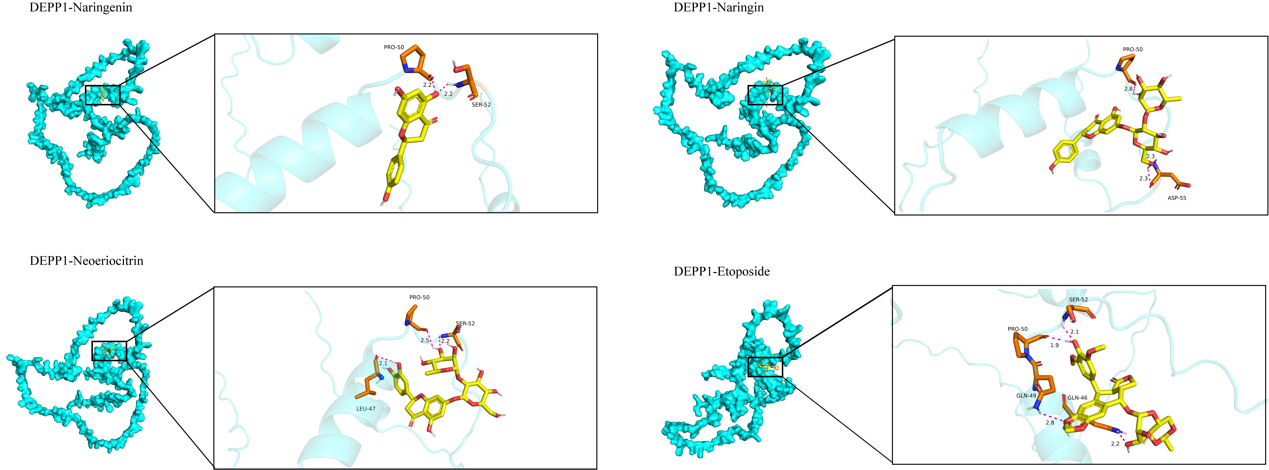


**Figure S4 Docking of TFDF constituents to DEPP1.**

Representative poses of naringenin, naringin, neoeriocitrin, and the reference ligand etoposide in the DEPP1 structure are shown with pocket close‑ups. All three TFDF flavonoids locate in shallow surface grooves of DEPP1 and form only sparse polar contacts (occasional H‑bonds) with mainly hydrophobic/π interactions, yielding weak predicted affinities (≈ −5.4 to −6.2 kcal mol⁻¹; see Table S3). Glycosides (naringin, neoeriocitrin) display highly dispersed, non‑convergent poses (large rmsd/ub, rmsd/lb), and etoposide, although more convergent, exhibits similarly low affinity. Together these results indicate poor compatibility of TFDF actives with DEPP1 and argue against DEPP1 as a direct binding target.


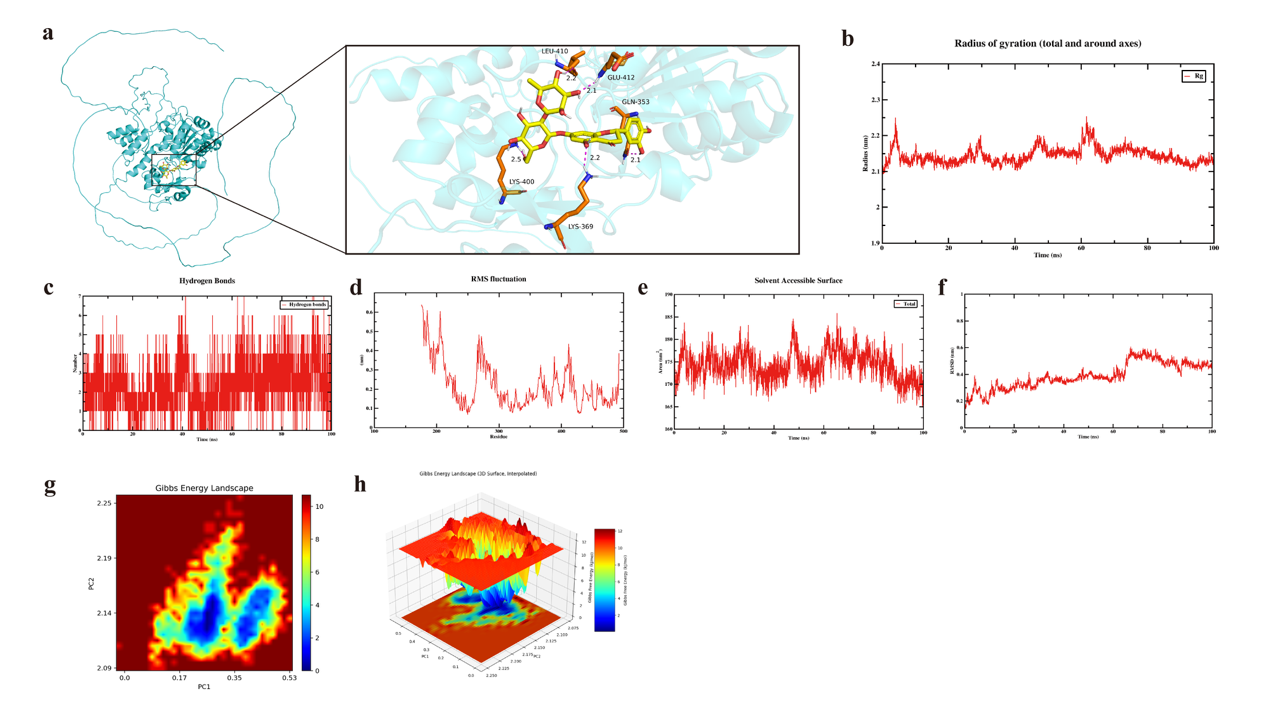


**Figure S5 MD analysis of the SIRT1–neoeriocitrin complex.**

(a) Molecular docking pose of neoeriocitrin in the SIRT1 activator pocket (key H-bonds/hydrophobics shown). (b) Radius of gyration (Rg) vs. time (ps); y-axis in nm. Rg decreases and stabilizes after ~10 ns, indicating that the protein becomes more compact upon binding. (c) Hydrogen bonds vs. time (ps); a steady number after ~10 ns indicates stable ligand–protein H-bonding. (d) RMSF per residue (nm); low global fluctuation with higher flexibility at the N-terminus. (e) SASA vs. time (ps; nm²); a decrease after ~10 ns suggests tighter solvent shielding around the bound ligand. (f) Ligand RMSD vs. time (ps; nm); stabilization after ~10 ns indicates a fixed pose within the binding site. (g h) Free-energy landscape of the SIRT1–naringenin complex (PC1 vs PC2), highlighting a dominant low-energy basin corresponding to the bound state.
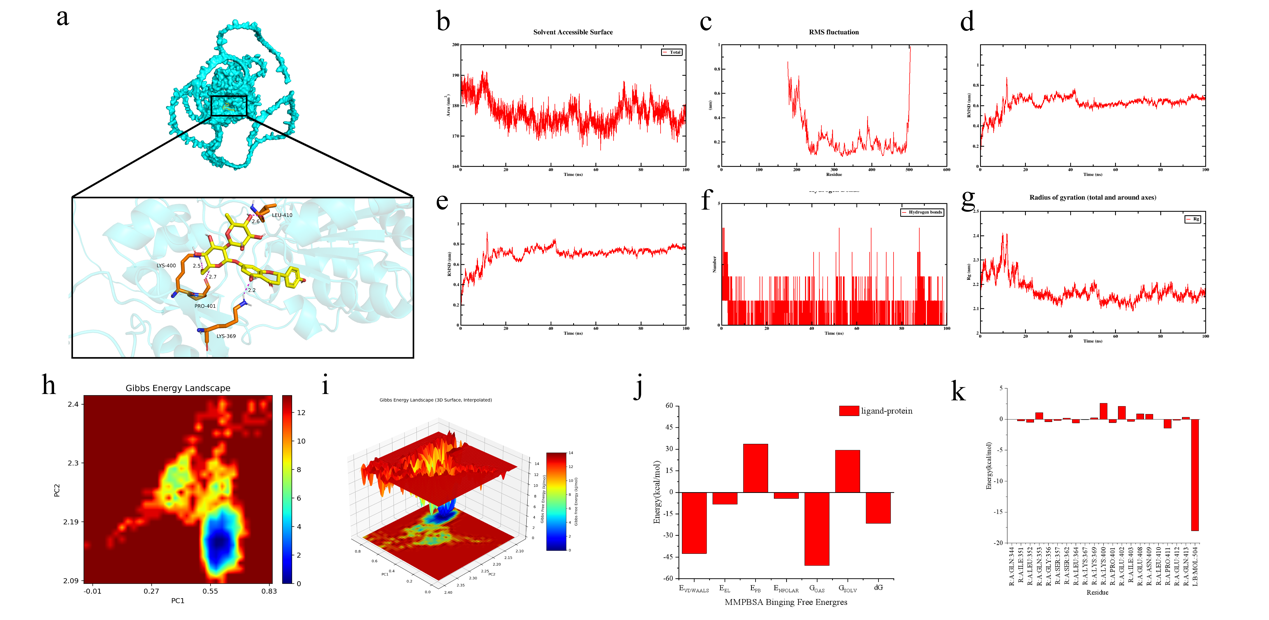


**Figure S6 MD analysis of the SIRT1–naringin complex.**

(a) Docked pose of naringin in the SIRT1 activator pocket with key H‑bond/hydrophobic contacts indicated. Along a 100‑ns simulation, stability metrics consistently supported a well‑behaved complex: solvent‑accessible surface area (b) and protein compactness (radius of gyration, d) declined and then plateaued after ~10 ns, indicating tighter packing around the ligand; backbone RMSD (c) reached a stable range after early equilibration; the ligand RMSD (e) equilibrated without drift, and a persistent 1–3 hydrogen‑bond network was maintained throughout (f). An additional Rg trace (g) showed the same convergence. Free‑energy landscapes (h,i) resolved a dominant low‑energy basin consistent with a single bound state. MM/PBSA decomposition (j) showed favorable van der Waals and electrostatic terms partially offset by polar solvation, yielding an overall negative binding free energy; residue‑level contributions (k) identified pocket residues as the major stabilizers. Together, these data indicate that naringin forms a stable, specific complex with SIRT1 over the full 100‑ns trajectory.


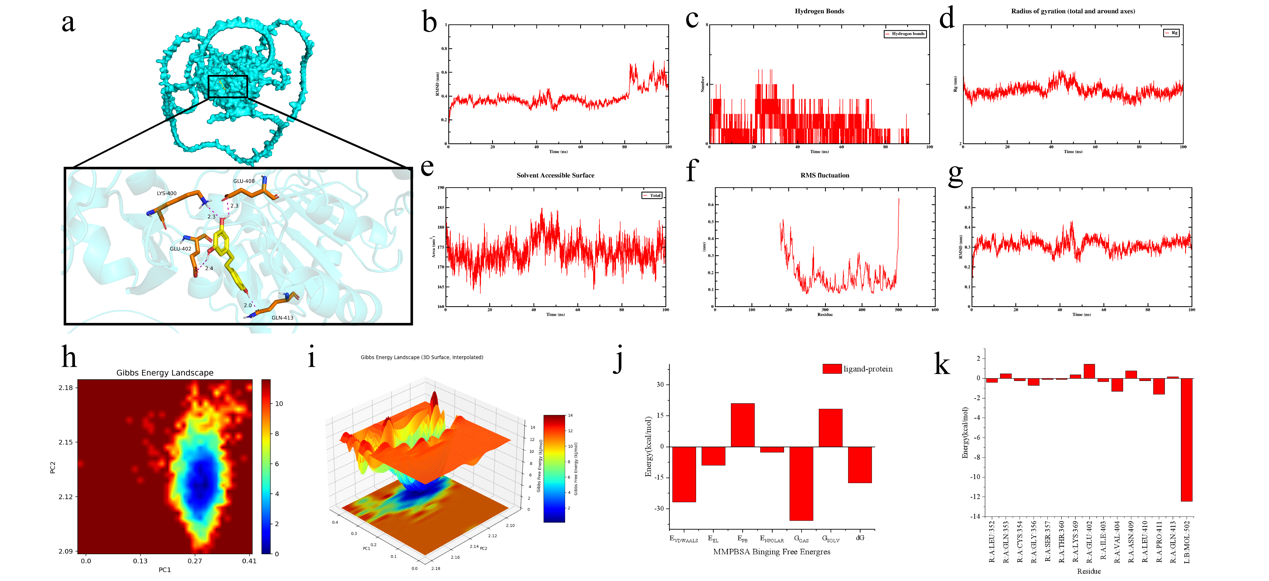


**Figure S7 SIRT1–resveratrol: docking and 100‑ns MD simulation**

Description. Panel a shows the docking pose of resveratrol within the SIRT1 activator pocket (AutoDock Vina binding affinity ≈ –6.9 kcal mol⁻¹), forming several hydrogen‑bond and hydrophobic contacts with pocket residues consistent with an activator‑like orientation. MD readouts across b–g indicate rapid equilibration and sustained stability: the radius of gyration and SASA reach steady plateaus after ~10 ns, the protein–ligand H‑bond trace remains persistent, RMSF is low for most residues except flexible termini/loops, and ligand RMSD shows no long‑term drift. The free‑energy landscapes (h,i) resolve a dominant low‑energy basin characteristic of a single bound state. MM/PBSA analysis (j,k) yields an overall favorable binding free energy (negative) with contributions mainly from hydrophobic residues lining the activator pocket, in agreement with the docking pose. Collectively, the trajectories support a stable SIRT1–resveratrol complex that serves as a positive‑control reference; stability is comparable though slightly weaker than the best TFDF constituents (see main text and Supplementary).


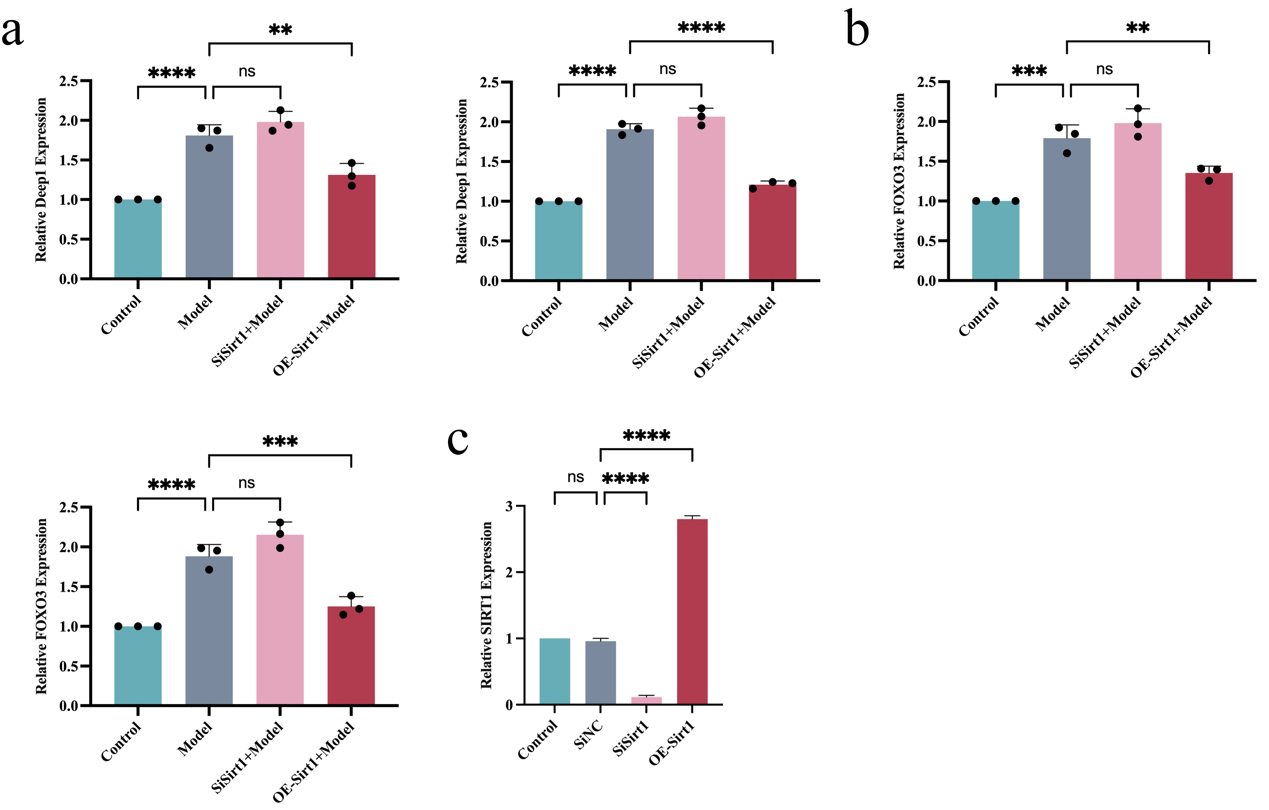


**Figure S8 SIRT1 dosage shapes stress‑responsive Depp1/Foxo3 transcription in neurons and osteoblasts.**
